# Disintegration and Machine-Learning-Assisted Identification of Bacteria on Antimicrobial and Plasmonic Ag–Cu_*x*_O Nanostructures

**DOI:** 10.1021/acsami.2c22003

**Published:** 2023-02-22

**Authors:** Furkan Sahin, Ali Camdal, Gamze Demirel Sahin, Ahmet Ceylan, Mahmut Ruzi, Mustafa Serdar Onses

**Affiliations:** † ERNAM—Erciyes University Nanotechnology Application and Research Center, Kayseri 38039, Turkey; ‡ Department of Electronic Engineering, Trinity College Dublin, Dublin 2 College Green, Dublin 2, Ireland; § Department of Biomedical Engineering, Yildiz Technical University, Istanbul 34220, Turkey; ∥ Faculty of Pharmacy, Erciyes University, Kayseri 38039, Turkey; ⊥ Department of Materials Science and Engineering, Erciyes University, Kayseri 38039, Turkey; # UNAM—Institute of Materials Science and Nanotechnology, Bilkent University, Ankara 06800, Turkey

**Keywords:** antibacterial, SERS, bacteria identification, bacteria detection, machine learning, silver nanoparticles, copper oxide nanoparticles

## Abstract

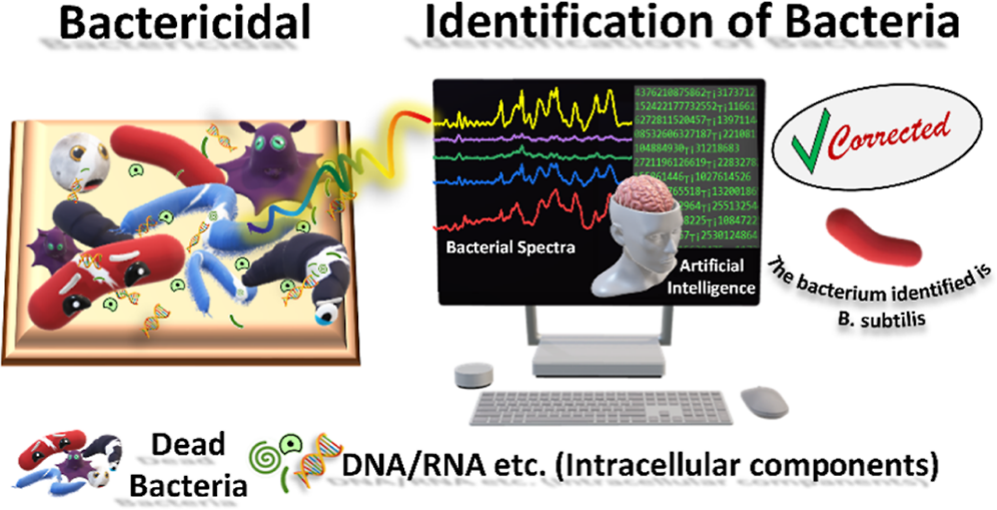

Bacteria cause many common infections and are the culprit of many outbreaks throughout history that have led to the loss of millions of lives. Contamination of inanimate surfaces in clinics, the food chain, and the environment poses a significant threat to humanity, with the increase in antimicrobial resistance exacerbating the issue. Two key strategies to address this issue are antibacterial coatings and effective detection of bacterial contamination. In this study, we present the formation of antimicrobial and plasmonic surfaces based on Ag–Cu_*x*_O nanostructures using green synthesis methods and low-cost paper substrates. The fabricated nanostructured surfaces exhibit excellent bactericidal efficiency and high surface-enhanced Raman scattering (SERS) activity. The Cu_*x*_O ensures outstanding and rapid antibacterial activity within 30 min, with a rate of >99.99% against typical Gram-negative *Escherichia coli* and Gram-positive *Staphylococcus aureus* bacteria. The plasmonic Ag nanoparticles facilitate the electromagnetic enhancement of Raman scattering and enables rapid, label-free, and sensitive identification of bacteria at a concentration as low as 10^3^ cfu/mL. The detection of different strains at this low concentration is attributed to the leaching of the intracellular components of the bacteria caused by the nanostructures. Additionally, SERS is coupled with machine learning algorithms for the automated identification of bacteria with an accuracy that exceeds 96%. The proposed strategy achieves effective prevention of bacterial contamination and accurate identification of the bacteria on the same material platform by using sustainable and low-cost materials.

## 1 Introduction

Contamination of surfaces with bacteria has become a serious problem in various areas of life, such as food packaging, medical implants, dentistry, and farming.^1^ Pathogenic bacteria transmitted from these contaminated surfaces can cause infections that threaten human health in both industrialized and developing countries, where more than 6.7 million people die each year due to bacterial infections.^2^ This problem particularly threatens low-income countries and infections caused by contamination are one of the major causes of death.^3^ The treatment of infectious diseases with antibiotics has become less effective as antibiotic-resistant strains have emerged. As a result, the treatment of bacterial infections has become progressively more difficult and new approaches are needed to combat this issue. Recently, researchers have been exploring alternatives, such as antimicrobial peptides,^4^ immune system mimetic artificial macrophages,^5^ reactive oxygen species (ROS) generating biocatalytic nanomaterials,^6,7^ and ion-releasing metallic nanocomposites^8^ as efficient non-antibiotic antibacterial strategies to combat bacteria. Most of these studies aim to kill the bacteria after they have interacted with the host cells. An attractive approach is to use antibacterial materials to disintegrate bacteria on host surfaces and prevent their transmission from the beginning.

The effective management of pathogen-related diseases is greatly improved by antibacterial surfaces and rapid, sensitive, and reliable bacteria detection techniques.^9^ Early detection of pathogens can prevent further spread and reduce transmission.^9,10^ Identification of the specific bacteria responsible for infection is crucial for formulating an effective treatment strategy. Traditional methods for detecting bacteria include staining, optical microscopy, microbial culture, and amplification techniques.^11^ In addition, new methods such as polymerase chain reaction and enzyme-linked immunosorbent assay are also used to detect low concentrations of bacteria.^12^ However, existing methods have some limitations, such as time-consuming and expensive sample preparation processes and sporadic false–positive results.

To address these limitations, researchers are currently developing simple, sensitive, and reliable methods for detecting and identifying pathogens. Advanced sensing technologies such as electrochemical detection,^13^ fluorescence,^14^ and Raman scattering^15,16^ are of interest. Raman spectroscopy, in particular, has gained tremendous attention for its ability to detect molecular vibrations with high sensitivity and rapid analysis. One inherent challenge of Raman spectroscopy is the weak inelastic light scattering of molecules, resulting in low intensities. Plasmonic nanostructures have been developed to overcome this challenge by significantly increasing the Raman scattering through electromagnetic enhancement mechanisms. Referred to as surface-enhanced Raman scattering (SERS), this approach enables detection of molecules at low concentrations, even down to a single molecule level. SERS has become one of the most promising techniques for meeting the demands of bacteria detection.^17−26^

Recent studies have focused on the development of SERS platforms for detecting a wide range of bacterial strains. Wang et al. developed a SERS platform by combining polyethyleneimine (PEI)-modified, Au-coated magnetic microspheres (Fe_3_O_4_@Au@PEI) with concentrated Au@Ag nanoparticles and reported a fast and sensitive detection of bacteria without any labeling.^27^ Using this platform, they were able to detect Gram-negative bacteria *Escherichia coli* and Gram-positive bacteria *Staphylococcus aureus* at a concentration as low as 10^3^ cells per milliliter within 10 min. Similarly, Yu et al. reported an antibacterial and SERS active nanocomposite prepared from MXene and Au nanoparticles for bacterial sterilization and detection.^28^ Using this multifunctional nanocomposite material, they achieved over 92% antibacterial activity against *E. coli* and *Bacillus subtilis* and identified these two bacterial strains through typical Raman bands of phospholipids, proteins, and polysaccharides.^28^ However, the number of common pathogens responsible for diseases is much greater and these bacteria also need to be identified with SERS. Liu et al. and Allen et al. have focused on this problem in their recent work and performed extensive bacterial detection with SERS.^29,30^ They observed that even though bacteria species can be identified from the SERS spectra for a small number of isolates, it becomes increasingly difficult when more bacteria species are investigated because the spectra appear to be similar.^30^ Therefore, traditional SERS spectra comparison methods are insufficient in practice, and advanced feature analysis techniques are needed.^31^ Machine learning techniques can aid in the feature extraction and comparison, as recently demonstrated by Rahman and colleagues, who were able to distinguish a large number of common bacterial strains with a high overall accuracy of 87.7%, revealing the potential of combining SERS biosensors with advanced analysis techniques.^31^ Nevertheless, almost all reported studies involve the transfer of bacterial suspension and mixing with colloidal plasmonic nanoparticles and transfer to a substrate for SERS measurements. Furthermore, most machine learning techniques used for SERS-based bacteria identification involves some data preprocessing steps, hampering fast and automatic classification. Therefore, there is a need for fast detection and identification of bacteria on surfaces using SERS coupled with machine learning techniques.

In this study, we present a multifunctional material platform for disintegration and detection of bacteria. The detection is achieved by Raman spectroscopy assisted by machine learning techniques for multiplex, rapid, and low-cost identification of common bacteria. Specifically, Ag–Cu_*x*_O nanostructures were developed by combining the excellent antimicrobial property of paper decorated with in situ grown Cu_*x*_O nanoparticles^32^ and flexible SERS surfaces^33^ on a single platform. The presented platform exhibited over 99% bactericidal properties and high SERS activity, allowing detection of bacteria at a concentration as low as 10^3^ cfu/mL. The disintegration of bacteria plays a key role in effective identification of bacteria. Additionally, the combination of this substrate with machine learning models enabled identification of several bacterial strains with high sensitivity, specificity, and accuracy that exceeds 96%.

## 2 Materials and Methods

### 2.1 Fabrication of Ag–Cu_*x*_O Nanostructures

A piece of print paper (1 × 3 cm^2^) was placed in a test tube to grow nanoparticles on it, followed by adding 15 mL of distilled water, 10 mg of silver nitrate (AgNO_3_ crystal, extra pure, Merck Millipore), and 25 mg of copper acetate [Cu(CO_2_CH_3_)_2_·H_2_O, Sigma-Aldrich]. Consequently, 3 mL of aqueous extract of *C. libani* was added. Here, the extract was prepared from *C. libani* wood, as detailed in our previous work. The polyphenols in the extract mediated the reduction of metal salts to form nanoparticles.^32^ Subsequently, the test tube was shaken continuously for 1.5 h at 95 °C in a water bath (Memmert WNB14) to allow for the growth of nanostructures on the paper. Afterward, the paper covered with nanostructures, was retrieved from the tube and left to dry at room temperature. For brevity, this sample is referred to as Ag–Cu_*x*_O nanostructures. For comparison, three more nanostructures were grown on a paper surface using only silver nitrate (10 mg), only copper acetate (100 mg), and four-fold increased concentration of the copper salt (mixture of 10 mg silver nitrate and 100 mg copper acetate).

### 2.2 Antibacterial Assay

The antibacterial activity of the samples was evaluated both qualitatively and quantitatively against Gram-negative bacteria, *E. coli* (ATCC25922), and Gram-positive bacteria, *S. aureus* (ATCT25923). Specifically, bacterial suspensions at 0.5 McFarland turbidity were prepared in a Mueller–Hinton broth. For qualitative analysis, the AATCC 147 parallel streak method was adopted. This analysis involved using a cotton swab that was dipped once into the prepared bacterial suspension and spreading on solid agar medium in parallel lines. The antibacterial activity of the samples (1 × 3 cm^2^) was evaluated qualitatively by measuring the inhibition zone diameter after 24 h of incubation at 37 °C and 85% humidity.

The bactericidal activity of the surface was evaluated quantitatively by following the AATCC 100 test protocol with a slight modification. Here, a 100 μL of the prepared bacterial suspensions was cultivated on the nanostructured surface. The samples were then kept in an incubator at 37 °C and 85% humidity for 24 h. After the incubation, the samples were immersed into 10 mL of PBS (phosphate buffer solution, ClearBand) and washed by sonication for 10 min and vortexing for 1 min. Consequently, a 100 μL of this suspension was fetched and spread on a solid agar plate using a glass Drigalski stick. After 24 h of incubation, the cell colonies formed on the agar plates were counted and the antibacterial activity value of the surfaces was calculated according to the following equation
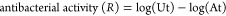
1At is the average number of colonies obtained from the fabricated nanostructures, while Ut is the average number of colonies obtained from the control samples. In similar standards, the critical threshold *R* value is recommended as 2, and if *R* ≥ 2, the material is considered as antibacterial.^34^

### 2.3 SERS Measurements

Raman measurements were performed using a confocal Raman microscope (Alpha 300 M+, WITec, Germany) with a laser wavelength of 532 nm. The SERS performance of the surface was evaluated by using rhodamine 6G (R6G, Sigma-Aldrich) as the probe molecule. Spectra were recorded by focusing the laser beam with a power of 1.5 mW on the sample surface with a 100× microscope objective (NA = 0.95) at an integration time of 0.5 s. The SERS activity of the nanostructures was evaluated by calculating the analytical enhancement factor (AEF) with the following equation^35^
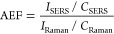
2Here, *C*_SERS_ (1 nM) and *C*_Raman_ (1 mM) are the concentrations of the R6G placed on the reference (Si wafer) and the nanostructured surface, respectively. *I*_SERS_ and *I*_Raman_ are the corresponding signal intensity at the peak of 1362 cm^–1^ in the measured spectra of R6G.

To collect the SERS spectra of various bacteria, suspensions containing 10^3^ cfu/mL bacteria (in Muller–Hinton broth) were washed three times to remove the impurities, followed by dispersing in PBS. Consequently, a 100 μL of bacterial solution in PBS was retrieved and spotted on the nanostructures and left to dry for 40 min. SERS spectra were recorded with a laser power of 10 mW and integration time of 0.5 s.

### 2.4 Identification of Bacteria by Machine Learning

To identify different types of bacterial species from the collected SERS spectra, we used the common machine learning algorithms from the open-source Python (3.8) library, Scikit-learn. To read, process, and visualize the spectral data, we used python packages: NumPy, SciPy, Matplotlib, and Seaborn.

To classify the five different bacteria species, 1114 SERS spectra were recorded on the Ag–Cu_*x*_O nanostructures. These include 157 for *Bacillus subtilis* (*B. subtilis*), 309 for *Escherichia coli* (*E. coli*), 155 for *Enterococcus faecalis* (*E. faecalis*), 343 for *Staphylococcus aureus* (*S. aureus*), and 150 for *Streptococcus mutans* (*S. mutans*). Specifically, the data were first normalized using StandardScaler and then principal component analysis (PCA) was applied on the transformed data. Machine learning methods were used to distinguish bacteria. To facilitate the machine learning-based identification for real-life adaptation, the spectral data obtained from bacteria were used directly, without any pre-processing such as background subtraction or smoothing. For each bacterial species, approximately 66.7% of the spectral data were used as training data, which was obtained by parsing it using the randomization parameter (randomization coefficient = 40) of the split function from the Scikit-learn library. These data were used to train classification algorithms like support vector machines (SVM), k-nearest neighbors (KNN), and decision tree. Finally, the remaining approximately 33.3% of the bacterial spectra were used to test the accuracy of the system.

### 2.5 Characterization

The chemical composition and morphology of the obtained surfaces were characterized using scanning electron microscopy (SEM, Zeiss EVO LS10), FE-SEM (field emission scanning electron microscopy) (Zeiss Gemini 500), and energy-dispersive spectroscopy (EDS, Bruker). Before imaging, a thin layer of gold was sputter-coated onto the samples. ImageJ software was used to determine the size distribution of the nanoparticles on surfaces from SEM images. The surface chemical composition of the nanostructures was analyzed using X-ray photoelectron spectroscopy (XPS, K-alpha, Thermo Scientific) with a monochromatic Al Kα X-ray source (1486.7 eV). Thin-film XRD analysis was performed with a diffraction meter (Panalytical Empyrean) operating at 40 kV and 30 mA using a Cu Kα radiation source. Finally, an FTIR microspectrometer (LUMOS II, Bruker) was used to analyze the IR spectrum of bacteria on the surfaces.

### 2.6 Investigation of the Bactericidal Mechanism of Ag–Cu_*x*_O Nanostructures: Ion Release and ROS Generation

The release of ions from the Ag–Cu_*x*_O nanostructures was assessed by immersing two surfaces (each measuring 1 cm × 1.5 cm) in separate beakers of 10 mL of deionized water. 2 mL of solution from each beaker was withdrawn at the end of the first and 24th hours. The concentrations of Ag and Cu ions were measured using inductively coupled plasma mass spectroscopy (ICP–MS, model 7500a, Agilent).

The production of ROS that causes the death of bacteria was evaluated on *E. coli* using 2′,7′-dichlorodihydrofluorescein diacetate (DCFH-DA, Cayman Chemical Company). DCFA-DA is a non-fluorescent probe that becomes highly fluorescent upon oxidation and is commonly used for sensitive and rapid detection of ROS. To detect ROS, 200 μL of *E. coli* (3 × 10^8^ cfu/mL) were cultivated on the surface of Ag–Cu_*x*_O. After 24 h of incubation, bacteria on the surface were collected by mixing them with 5 mL of PBS using sonication and vortexing. As a control, untreated *E. coli* (200 μL of 3 × 10^8^ cfu/mL) was also collected using the same method. The suspensions were washed three times by centrifugation at 4000 rpm for 3 min. Next, the bacteria were stained with DCFA-DA (final concentration of 100 μM) and incubated in the dark at 37 °C for 30 min. Afterward, the samples were washed with PBS twice, followed by placing 10 μL of the stained bacterial suspension between two glass slides (20 mm × 20 mm). A microscope (ZEISS Axio Imager 2) with a Filter 38 set (BP470/40 excitation filter and BP525-550 emission filter) was used to take the fluorescent images of the bacteria and observe the production of ROS.

## 3 Results and Discussion

### 3.1 Preparation and Characterization of Ag–Cu_*x*_O Nanostructures on Paper

**Figure 1:**
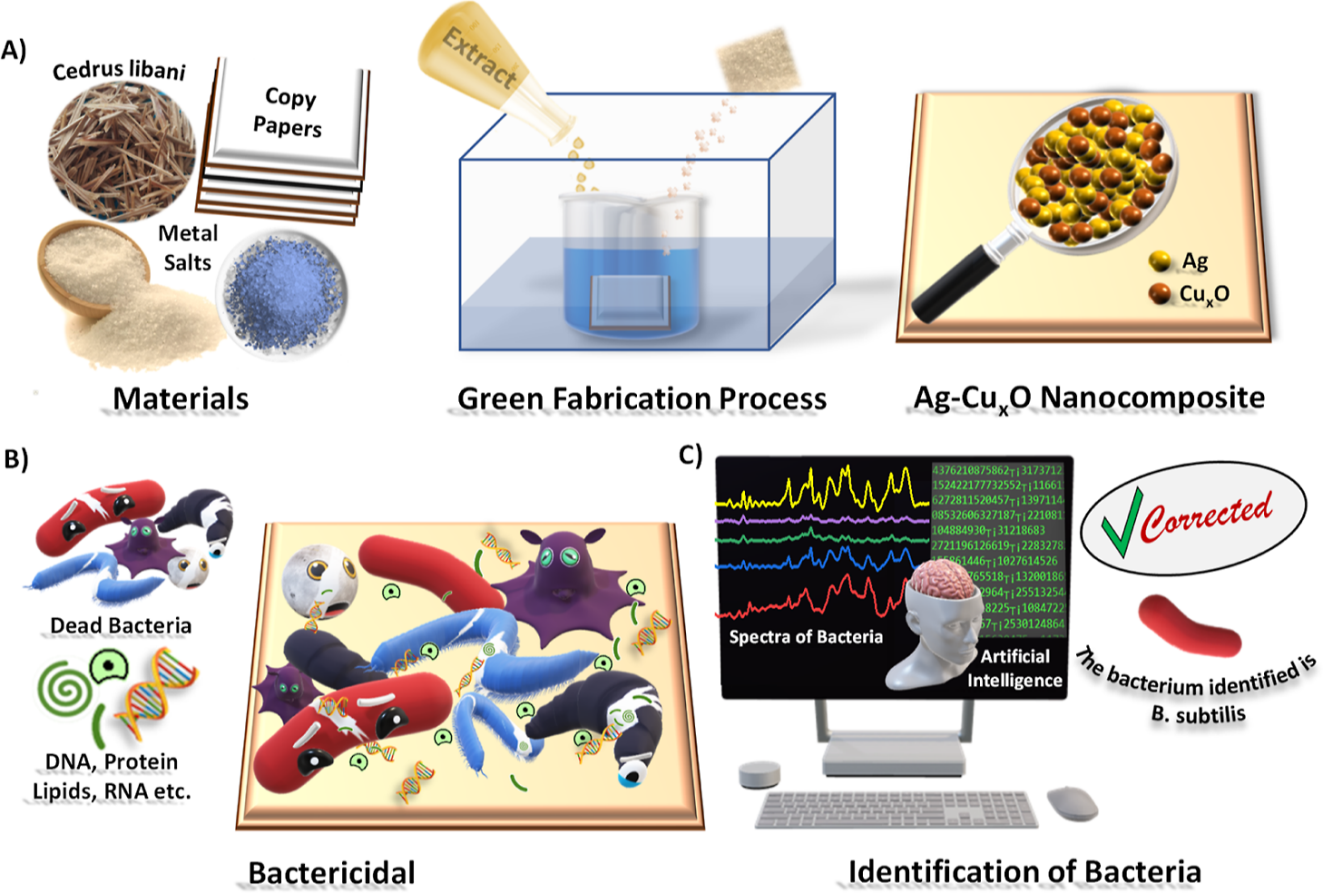
Schematic illustration of (A) materials used and the steps involved in fabricating the Ag–Cu_*x*_O nanostructures, (B) disintegration of bacteria on the Ag–Cu_*x*_O nanostructures, and (C) detection and identification of bacteria through the collected SERS spectra and classification using machine learning.

The functionalization of paper surfaces with copper oxide and silver nanoparticles was carried out in a single step via an in situ growth method, using only three materials (Figure 1a). Instead of expensive chemicals, an aqueous extract of naturally collected *C. libani* plant (Figure S1A), a piece of copy paper, and metal salts were used. These materials were placed in a container and heated in a water bath at 95 °C for 90 min. This process results in the growth of Ag–Cu_*x*_O nanostructures on the micro-structured surface of the paper, presumably via the reduction of salt ions by the polyphenols present in the *C. libani* extract.^32,33^ As a result, the color of the paper changes from white to gray (Figure S1B). Furthermore, the produced surface also exhibits high antibacterial activity against Gram-negative and Gram-positive bacteria and can disintegrate the bacteria on the surface (Figure 1b). Additionally, the electromagnetic enhancement provided by the nanostructured surface enables SERS-based identification of bacterial strains. When combined with machine learning, this SERS capability exhibits high accuracy (96%) for detecting and identifying surface-contaminating bacteria. The inexpensive (∼$0.16, Table S1), fast, and label-free platform shows great promise for use in a wide variety of fields for screening bacteria.

**Figure 2:**
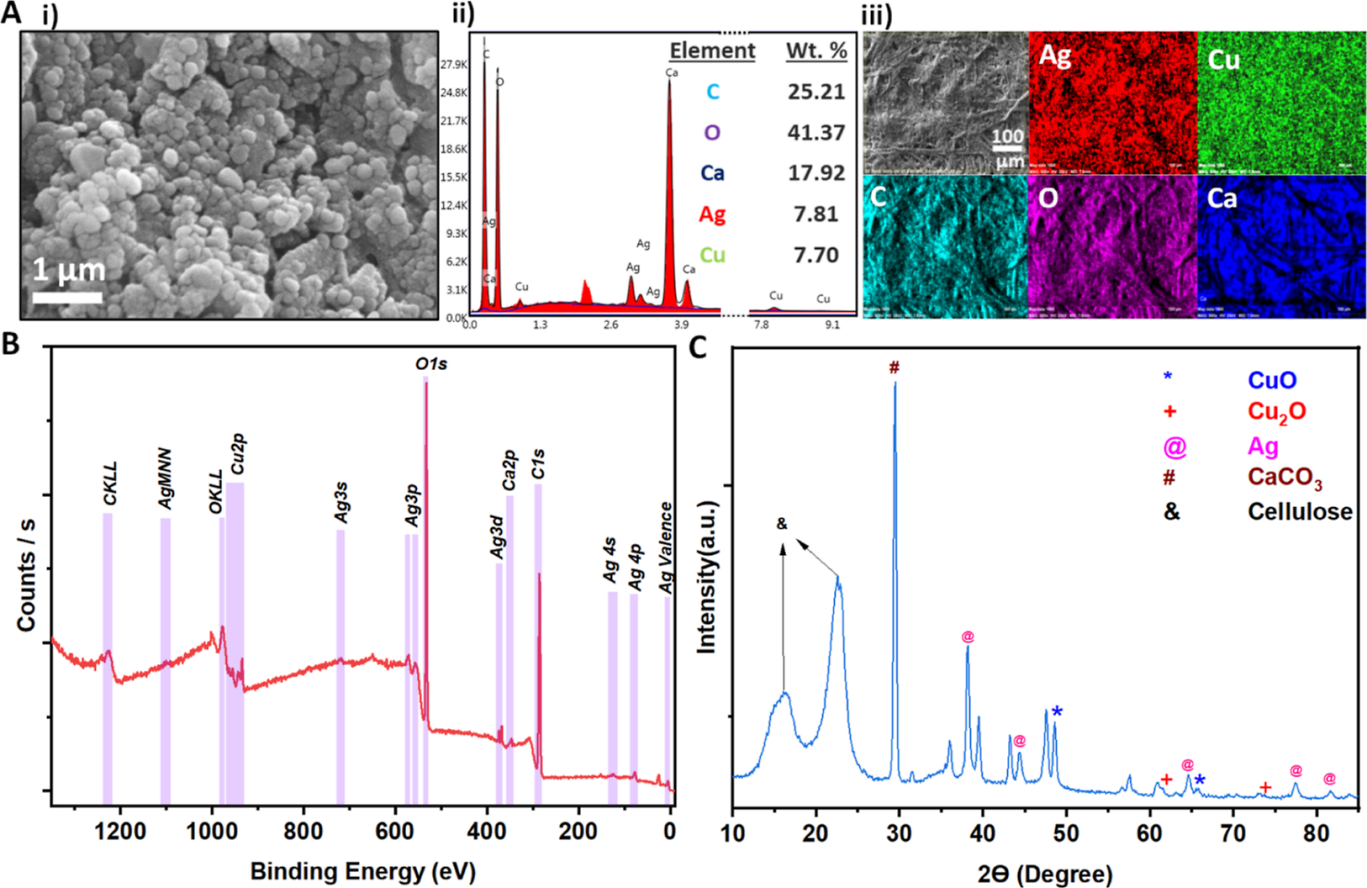
Chemical and structural characterization of the Ag–Cu_*x*_O nanostructures. (A) SEM images (magnification of 25,000) (i), EDX elemental analysis (ii), and elemental mapping (iii) of Ag–Cu_*x*_O nanostructures at a magnification of 500. (B) XPS survey scan and (C) XRD pattern of Ag–Cu_*x*_O nanostructures and assignment of some major peaks. For detailed assignments, see Supporting Information, Table S2.

The structure and composition of the grown Ag–Cu_*x*_O nanostructures were examined using various techniques. SEM images show that the nanostructures are made of aggregates of spherical nanoparticles with a diameter of 109 ± 50 nm (Figures 2a and S2). EDX analysis confirms the presence of elemental silver (7.81%) and copper (7.70%) on the surface, and EDX elemental mapping implies homogeneous distribution of the nanoparticles. To elucidate the chemical nature of surface species, further characterization was performed using XPS. As shown in Figure 2b, the XPS survey spectrum consists of characteristic peaks of Cu 2p, Ag 3d, O 1s, and C 1s. The high-resolution XPS spectrum around the Cu 2p region consists of main peaks at 933.5 eV (Cu 2p3/2) and 953.9 eV (Cu 2p1/2), convolved with the respective shake-up satellite peaks (Figure S3A). The peaks suggest that the copper nanoparticles on this surface are primarily oxidized copper species such as Cu_2_O. The high-resolution XPS scan around the Ag 3d region indicates that silver is in the Ag^0^ metallic state (Figure S3B). These results are important for understanding the antibacterial activity of oxidized copper and the SERS effect of metallic silver (Ag^0^).

The binding energies of Cu(I) and Cu(II) are very close to each other, so the composition of copper nanostructures could not be clearly identified by XPS analysis. Therefore, further characterization is conducted using XRD, as shown in Figure 2c and Table S2. First, the peaks observed at 2θ = 32.6, 35.7, 48.8, 53.4, 58.2, 61.6, 65.8, 66.2, and 68.1 correspond to the (110), (002), (1), (2), (020), (220), (3), (022), (1), and (220) planes of the CuO (JCPDS nos. 05-0661), respectively.^32^ Furthermore, the existence of Cu_2_O is confirmed via the XRD peaks observed at 2θ = 61.51, 73.75, and 77.53° (JCPDS cards nos. 75-1531 and 05–0667).^32^ The XRD analysis also confirms the existence of metallic Ag^0^ via peaks located at 38, 44, 65, 77, and 82°, corresponding to planes (111), (200), (220), (311), and (222) of the fcc (face-centered cubic) crystal structure (JCPDS, file nos. 04-0783), respectively.^36^ Besides these, peaks originating from the paper (cellulose and CaCO_3_) substrate also show up in the XRD (elemental analysis of the untreated paper is shown in Figure S4).

### 3.2 Evaluation of the Antibacterial Activity of Ag–Cu_*x*_O Nanostructures

**Figure 3:**
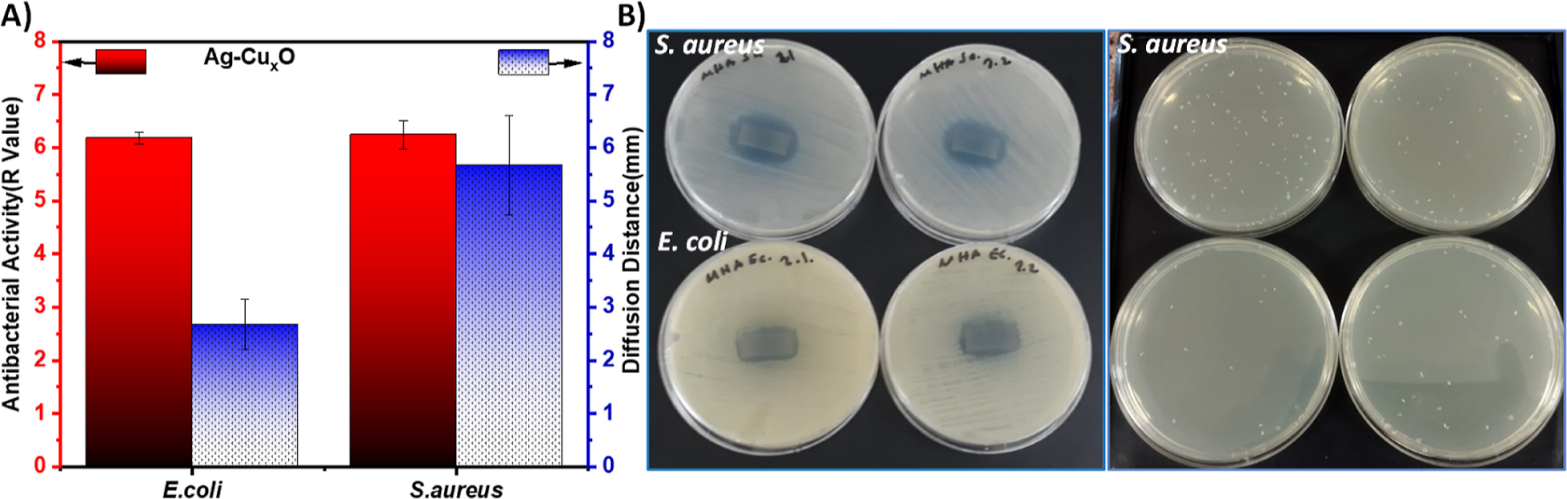
Antibacterial activity of the Ag–Cu_*x*_O nanostructures grown on the paper. (A) The bactericidal activity and diffusion distances. (B) Photographs of agar plate showing the diffusion disk results (left) and photographs of agar plate showing the killing test (right).

The antibacterial activity of Ag–Cu_*x*_O nanostructures was evaluated using Gram-positive bacteria *S. aureus* and Gram-negative bacteria *E. coli*. As shown in Figure 3 (also Table S3), the Ag–Cu_*x*_O nanostructures inhibited the growth of bacteria with inhibition diameters of 2.33 mm for *E. coli* and 4.83 mm for *S. aureus*. Concerning bactericidal activity, the Ag–Cu_*x*_O nanostructures killed almost all bacteria, while bacteria on untreated surfaces increased by approximately ∼200 times after only 24 h (Table S4). Overall, the nanostructured surface had very high antibacterial activity against both types of bacteria (99.9999%, *R* value > 6) and was more effective in inhibiting the Gram-positive bacteria.

Both copper oxide and silver are commonly used as antibacterial materials and further evaluation is needed to determine the main reason for their high antibacterial activity. To investigate the effect of copper oxide on the antibacterial activity, we measured the growth inhibition ability and bactericidal efficacy of the nanostructures composed of solely Ag and Ag–Cu_*x*_O prepared by increased concentration of the copper acetate salt (mixture of 10 mg of silver nitrate and 100 mg of copper acetate). The bactericidal activity of the different nanostructures increased with the amount of copper salt: the *R* values for *E. coli* are 5.68, 6.18, and 6.92 and for *S. aureus* are 5.95, 6.24, and 6.95 for the nanostructures prepared by using none, 25, and 100 mg of copper acetate, respectively (Table S4). Similarly, the Ag nanostructure without any copper had approximately a 3 times smaller zone of inhibition diameter for *E. coli* and no inhibition at all for *S. aureus* (Figure S5 and Table S3). Furthermore, the Ag–Cu_*x*_O composite surface can deactivate bacteria very quickly, even at a high bacterial concentration of 3 × 10^8^ cfu/mL, only within 30 min, while it takes more than 3 h for the Ag nanostructures to achieve the same bactericidal activity (Figure S6). These results indicate that copper oxide is the main source of the antibacterial activity of the Ag–Cu_*x*_O nanostructures. This result is consistent with the findings of a previous study which demonstrated that copper has a stronger antibacterial effect than silver.^37^ Here, the significantly higher bactericidal activity of copper oxides is likely due to the high ion release rate of metal oxides (Cu^+2^ and Cu^+^ for this study).^38−40^ The ion release measurements of Ag–Cu_*x*_O (Table S5) also support these findings. High concentrations of ions (especially copper) bind to both the inner and outer parts of the bacterial cell membrane, lipopolysaccharides, peptidoglycans, and carboxylic groups, reducing the potential difference between intracellular and extracellular components, causing depolarization and instability in the cell membrane.^41^ The result is the rupture of the cell membrane and disintegration of bacteria.^42^ Additionally, metallic nanoparticles can generate ROS that induces cellular oxidative damage by causing DNA/RNA breakage, protein oxidative carbonylation, membrane disruption, and lipid peroxidation, eventually leading to the death of microorganisms.^43^ This hypothesis was supported by in vitro detection of green fluorescence emission of internalized DCFH-DA, a fluorogenic marker that is sensitive to ROS (Figure S7). Therefore, the Ag–Cu_*x*_O sample produces ROS that can contribute to the bactericidal property.

### 3.3 SERS Activity of the Ag–Cu_*x*_O Nanostructures

**Figure 4:**
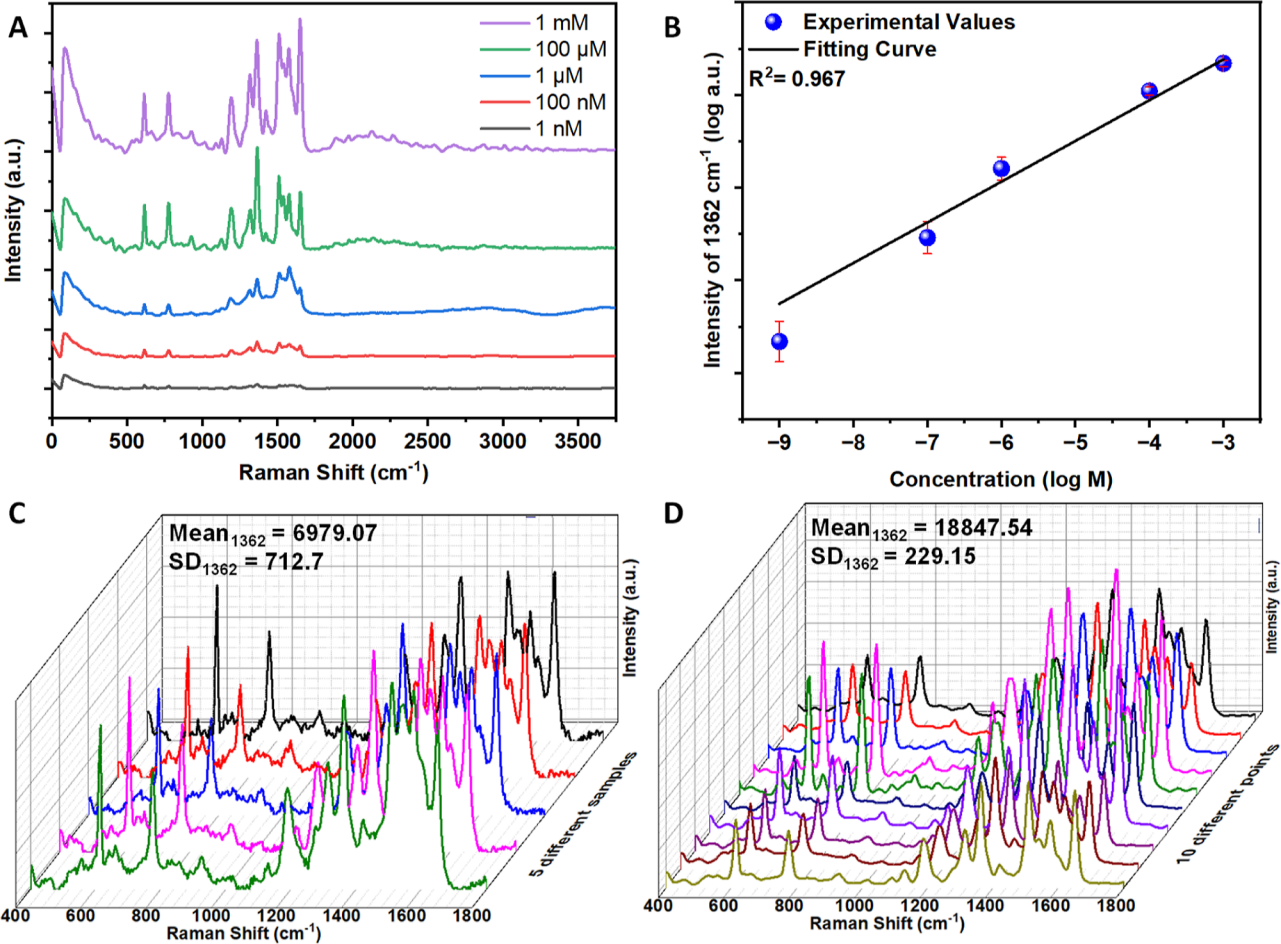
SERS activity of the Ag–Cu_*x*_O nanostructures using R6G as the analyte. (A) SERS spectra of R6G molecule measured at various concentrations, as indicated in the legend. (B) The SERS intensity of the peak at 1362 cm^–1^ as a function of R6G concentration, and a fitting (*R*^2^ = 0.97). (C) Demonstration of sample-to-sample reproducibility. Shown are SERS spectra of the R6G analyte (concentration 100 μM) recorded from the surfaces of five different samples. (D) SERS spectra of the R6G analyte (concentration 100 μM) recorded from 10 different spots on the same sample, demonstrating spot-to-spot reproducibility (SD stands for standard deviation).

The surface of Ag–Cu_*x*_O nanostructures exhibits excellent antibacterial activity and thus is promising for practical applications to avoid the risk of fomite contamination. However, it is also important to be able to detect and identify these pathogens, especially during outbreaks. The presence of metallic silver in the prepared nanostructure prompted us to exploit these structures in the SERS-based detection of bacterial pathogens. For this purpose, we first characterized the SERS activity of the nanostructured surface using R6G as a probe molecule and studied the detection limit, enhancement factor, repeatability, and analyte concentration-SERS intensity relationship. As shown in Figure 4, the characteristic peaks of R6G^36,44^ are observed at 614, 773, 1187, 1315, 1364, 1512, and 1649 cm^–1^ and are clearly visible down to 1 nM concentration. It should be noted that no Raman spectra could be collected on the untreated paper surface, even at a very high concentration (Figure S8A). The composite nanostructure showed a high level of SERS activity with an AEF of 5.1 × 10^6^ (Figure S8B), which is comparable to other studies.^45,46^ Additionally, a linear relationship was found between SERS intensity and R6G concentration (Figure 4b), with a coefficient of determination (*R*^2^) value of ∼0.97.

Reproducibility is an important factor for using SERS platforms in practical applications. Figure 4c shows the R6G spectra recorded on five different substrates with the grown Ag–Cu_*x*_O nanostructures. Here, the standard deviation between samples is ∼10%, indicating excellent sample-to-sample reproducibility.^47^ Additionally, the similarity of SERS spectra recorded from randomly chosen 10 points indicates point-to-point uniformity. It should be noted that SERS activity is due to silver in the nanostructures. To confirm this finding, we fabricated copper oxide surfaces without silver which showed no SERS activity (Figure S9). It is also important to note that surfaces with only silver show a much higher SERS effect.^33^ Furthermore, it seems that the incorporation of copper oxide for high antibacterial activity reduces the SERS activity (Figure S9). The strong antibacterial activity of copper oxide indirectly contributes to the detection of bacteria through leakage of intracellular components. Overall, the surface of the Ag–Cu_*x*_O nanostructures strikes a balance between rapid antibacterial properties and high SERS activity, making it ideal for detecting and identifying various pathogenic bacteria species on the same platform.

### 3.4 Analysis of Bacteria with SERS

In this section, we study the detection of bacteria at low bacterial loads using the SERS characteristic of the fabricated nanostructures. For most bacterial species, a critical threshold of 1 × 10^5^ cfu/mL is considered an optimal sign of infection in the body.^48^ Thus, systems that detect bacteria must meet this minimum critical level of detection. Encouraged by the high SERS activity of our Ag–Cu_*x*_O nanostructures, we recorded SERS spectra of five different bacterial strains, *B. subtilis*, *E. coli*, *E. faecalis*, *S. aureus*, and *S. mutans*, at a concentration of 10^3^ cfu/mL on the Ag–Cu_*x*_O nanostructures. It should be noted here that a total of five species including four strains of Gram-positive bacteria were selected to assess the detection and identification among bacterial strains.

**Figure 5:**
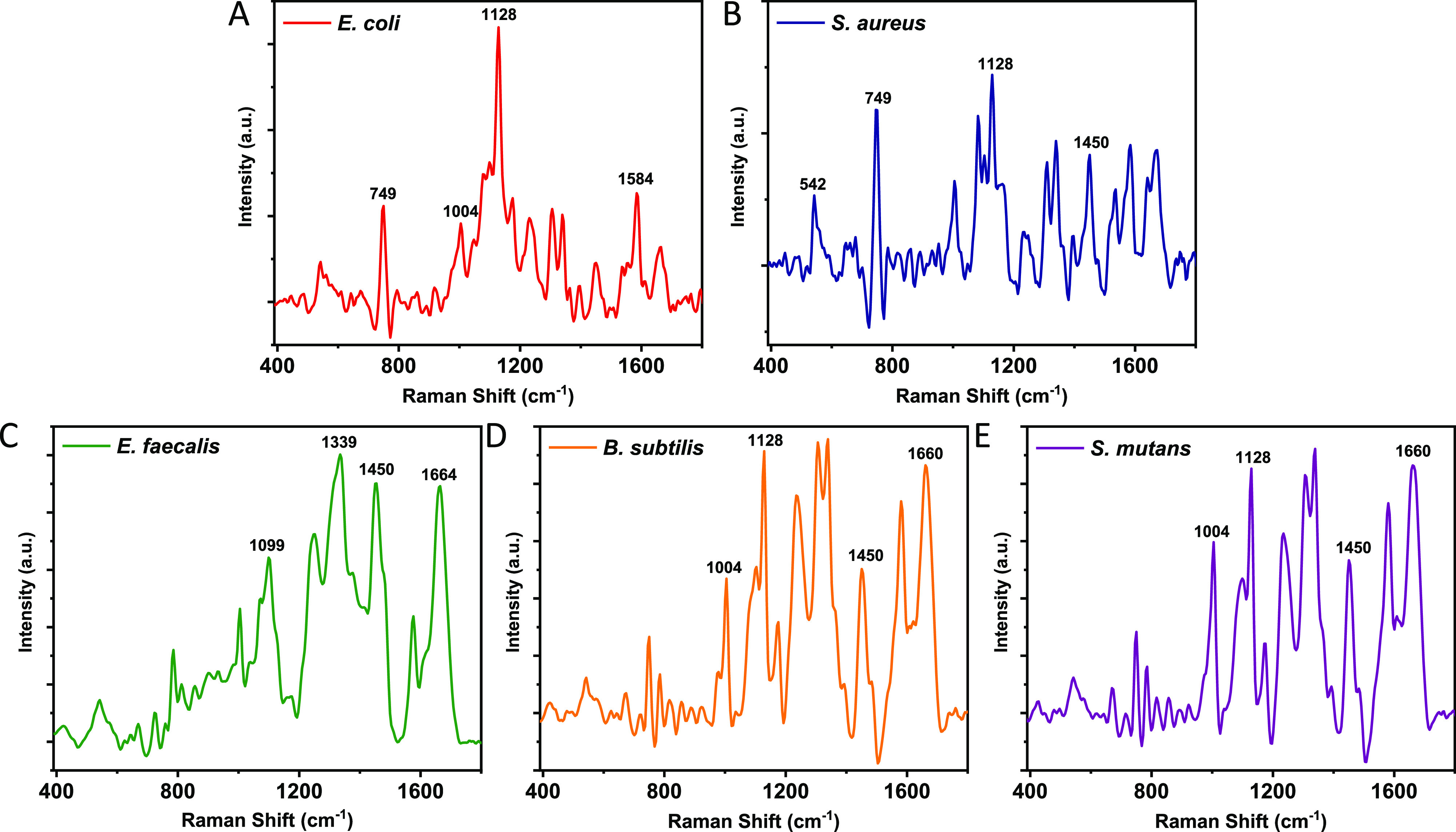
SERS spectra of bacterial species recorded on the Ag–Cu_*x*_O nanostructures. (A) *E. coli*, (B) *S. aureus*, (C) *E. faecalis*, (D) *B. subtilis*, and (E) *B. subtilis*.

Shown in Figure 5 are the SERS spectra of the bacteria species in the fingerprint region. The significant peaks are labeled and assigned (Tables 1 and S6). These peaks originate from carbohydrates, lipids, nucleic acids DNA and RNA, proteins, and amino acids. The 3060–3090 cm^–1^ peaks assigned to the stretch vibrations of heteroaromatic groups (Figure S10),^49^ the 2882, 2933, and 3060 cm^–1^ peaks assigned to the C–H stretch vibrations and the 1450 cm^–1^ peak assigned to CH_2_ bending vibration associated with proteins and fats^19,50−55^ and are strongly included in the spectra of all bacterial species. Ring breathing and ring stretching vibration modes associated with the five main nucleobases (adenine, guanine, thymine, uracil, and cytosine) in the nucleic acids are observed at 673, 785, and 1580 cm^–1^.^51,53−56^ Amino acids such as tryptophan (C–H bending peak at 1339 cm^–1^) and phenylalanine (the C–C aromatic ring breathing mode at 1004 cm^–1^) and typical amide I and amide III bands of proteins are also observed at 1230–1247 and 1660 cm^–1^.^19,51−56^ Each bacteria species showed multiple complex peaks some of which seem to be unique (Figure 5 and Table S6). It should be noted here that the differences in all these spectra are due to the Ag–Cu_*x*_O nanostructures because the spectra of bacteria collected on a glass slide look similar (Figure S11). As a result, it is possible to collect bacterial spectra at low concentrations compared to previous studies,^57−60^ but further analysis of the spectra is needed for bacterial identification.

**Table 1:** Assignment of the Most Significant Peaks in the SERS Spectra of Bacteria^a^

E. coli	S. aureus	E. faecalis	B. subtilis	S. mutans	assignment	compound
673	678	669	673	669	ring vibrations	T, G^54,56^
749	749	758	749	749	ring breathing	Trp, T^51,52,56^
794	785	785	785	785	υ(O–P–O), ring breathing	DNA, C, T^53−56^
1004	1004	1004	1004	1004	C–C ring breathing	Phe^52−54^
1099	1103	1099	1103	1099	υ(C–O), υ(C–C), υ(–C–OH), O–P–O^–^	carbohydrates, DNA^53,54,56^
1128	1128	1124	1128	1128	υ(C–N), υ(C–C), υ(C–O–C)	amide III, A, Phe^19,51,53,54^
1230	1230		1234	1234	υ(C–C), δ(C–C)	amide III^51,53,55,56^
	1247	1247			δ(CH_2_), υ(C–C), δ(C–C)	amide III, amide I, C, A^19,51−54,56^
1305	1310	1310	1305	1305	–C–H def	A, protein^53,56^
1339	1339	1339	1339	1339	–CH deformation, υ(NH_2_), δ(C–H)	A, G, DNA, Trp^19,51,53,54,56^
1450	1450	1450	1450	1450	–CH_2_ deformation, δ(CH_2_)	proteins, saturated lipids, Trp^19,51−55^
1584	1584	1576	1580	1580	ring stretch	A, G, Tyr^51,53−56^
1664	1668	1664	1660	1660	υ(C=O), υ(C=N), δ(NH_2_)	amide I, T, C^19,54,56^

a υ means stretching, δ is in-plane bending and γ means out-of-plane bending. Abbreviations: A, adenine; G, guanine; T, thymine; C, cytosine; Tyr, tyrosine; Phe, phenylalanine; Trp, tryptophan.

It is worth noting that the SERS spectra of the bacterial species indicated high levels of intracellular components, probably due to the antibacterial property causing death and leakage of cellular components. This observation suggests that the bacteria were disintegrated when cultivated on the Ag–Cu_*x*_O nanostructures, which was confirmed by measuring the FTIR spectrum of *E. coli* cultivated on the Ag–Cu_*x*_O nanostructure surface (Figure S12). Specifically, amide I (at 1647 cm^–1^) and amide II (at 1541 cm^–1^) bands originating from proteins and nucleic acids are clearly visible in the FTIR spectrum.^61^ Furthermore, the peak at 1238 cm^–1^ is associated with asymmetric phosphate stretching and shows that DNA/RNA components are revealed by the antibacterial effect of the surface.^61^ Similarly, the bactericidal tests show approximately four logarithmic reductions of *E. coli* concentration in just 1 h (Figure S13), indicating fast bacterial disintegration.

One of the most important factors in identifying bacteria is the reproducibility of their spectra. To evaluate the reproducibility, we measured the SERS spectra of *E. coli* cultivated on the surface after 1 and 24 h (Figure S14A). The spectra are broadly similar, indicating that the platform is suitable for detecting bacteria and there is no significant incubation time-dependent interference. However, there are some minor differences in the intensity. After 24 h, the intensity of peaks associated with tryptophan, adenine, and guanine (749, 1128, and 1584 cm^–1^) decreased, while peaks associated with DNA, tyrosine, and phosphate groups (781, 856, and 1640 cm^–1^) increased. Similarly, the spectra recorded from different parts of the surface are similar (Figure S14B). For this purpose, SERS signals collected from six different areas, with a total size of 288 μm^2^ are presented in Figure S14B. The relative standard deviations are 14, 14, 16, 14, 18, and 12%, for peaks positioned at 749, 1004, 1128, 1305, 1335, and 1584 cm^–1^, respectively. The result shows that the surface provides reproducible signals at a level of the state-of-the-art SERS substrates, with a relative standard deviation of less than 20% for bacteria. In summary, there are only minimal changes in SERS signals within a 24 h period and among different spots, demonstrating that the proposed platform can be used to identify surface-contaminating bacteria.

An additional characteristic that is important for the detection of bacterial contamination is sensitivity. To probe the sensitivity, SERS spectra of *E. coli* were recorded at concentrations ranging from the critical threshold of 1 × 10^5^ cfu/mL to 1 × 10^2^ cfu/mL. The platform can distinguish the characteristic peak at 1128 cm^–1^ associated with *E. coli*, down to 10^2^ cfu/mL (Figure S15A). However, at this low concentration, it can be clearly seen that the signal approaches to noise, so the ideal detection limit accepted for the developed platform was determined as 10^3^ cfu/mL. The SERS signals of bacteria grown on Ag–Cu_*x*_O nanostructures decreased with the concentration of bacteria. Here, the dependence of the SERS intensity of the Raman signal on bacteria concentration can be represented using a linear equation (*y* = α*x* + β, where α = 0.52 ± 0.1 and β = 0.87 ± 0.36) in logarithmic scale with a correlation coefficient of 0.93 (Figure S15B). These results show that the bacterial concentration can be inferred from the SERS intensity and reveals the sensitivity of the prepared platform.

### 3.5 Identification of Bacteria with Machine-Learning-Assisted SERS Analysis

**Figure 6:**
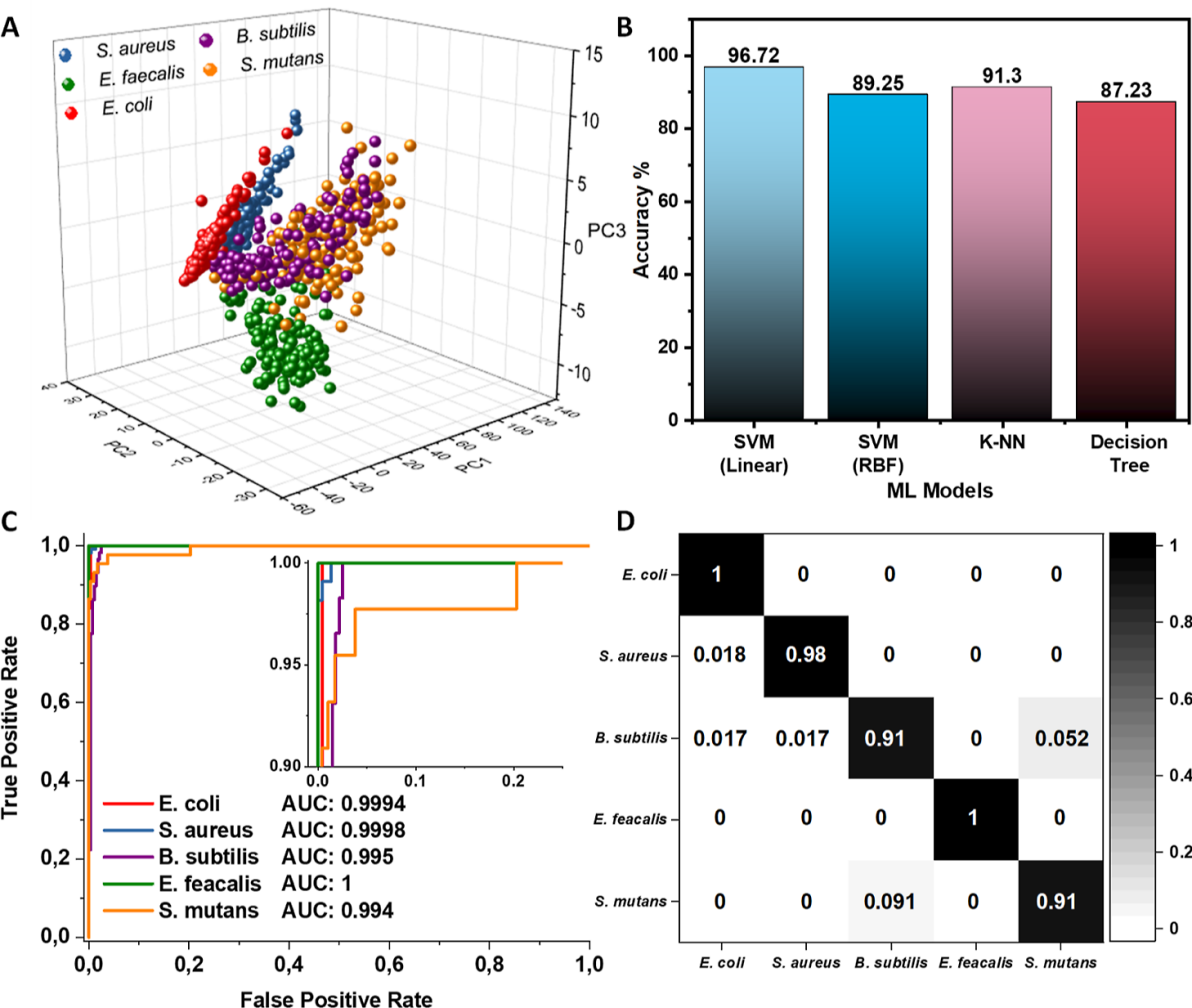
Machine learning classification of the five bacterial species from the SERS spectra recorded from Ag–Cu_*x*_O nanostructures. (A) 3D plot of the principal components. (B) Classification accuracies obtained from different machine learning models. (C) ROC curves for five different bacteria from the linear kernel of SVM classification. (D) Confusion matrix of the *B. subtilis*, *E. coli*, *E. faecalis*, *S. aureus*, and *S. mutans* prediction using the linear kernel of SVM analysis.

Identification of bacteria using SERS spectra is a difficult task. To overcome this challenge, we resort to machine learning to identify and classify different bacterial strains. For classification, the spectral data are first processed via PCA to preserve most of the information while significantly reducing the data dimensions. After PCA, each spectrum was then reduced to three key features. Shown in Figure 6a is the 3D PCA score space. Each bacterial class is clustered at a central point within itself, making it easy to see how different types of bacteria are separated from one another. For example, *E. coli*, *E. faecalis*, and *S. aureus* are well separated from *S. mutans* and *B. subtilis*. Additionally, individual planes can aid in identifying different bacteria. For example, the PC1-PC2 plane can be used to identify *S. aureus*, the PC2-PC3 plane for *E. faecalis*, and the PC1-PC3 plane for *E. coli* (Figure S16). The loading plots show (Figure S17) the principal components and how they contribute to the overall variance of the data. For example, the loading plot of PC1 explains 83% of the variance in the spectra and includes main peaks at 749, 1004, 1128, 1339, 1450, 1584, and 2933 cm^–1^. These results confirm that the PCA-reduced data are successful in preserving the important information and that the grouping is due to differences in their Raman signals.

The PCA results combined with SERS spectra of bacteria showed that three bacterial species can be easily identified without the need to examine Raman bands in detail. However, it was not possible to distinguish between *S. mutans* and *B. subtilis* using PCA and therefore advanced classification models are needed. 1114 measurements (157 *B. subtilis*, 309 *E. coli*, 155 *E. faecalis*, 343 *S. aureus,* and 150 *S. mutans*) were collected from five different bacterial species and were randomly divided into train and test sets at rates of 66.7 and 33.3%, respectively. These data were used as inputs for SVM, KNN, and Decision Tree machine learning models. As shown in Figure 6b, the linear kernel of SVM was found to classify the data set better than the SVM RBF kernel, KNN, and Decision Tree, with an accuracy of about 97%. Moreover, the linear core of the SVM exhibits over 95% accuracy for each set when trained with 50 randomly distributed training sets, with an average accuracy of 97.2 ± 0.9%, resulting from 50 sets (Figure S18). This high performance is likely due to the linearity of the data set and the fact that each SERS spectrum contains 1024 features. These results are similar to another study, which used SVM-assisted SERS to detect bacteria at the level of 10^3^ cfu/mL and identify 19 different bacterial species with an accuracy of 87.7%.^31^Figure 6c shows the prediction accuracy of the SVM algorithm for each bacterium using the area under the ROC curve. Accordingly, the curve for *E. faecalis* shows no false positives, and the curves for *E. coli* and *S. aureus* show very little false positive information with the area under the curve close to ∼1. Similarly, though it exhibits more false positives for *B. subtilis* and *S. mutans*, the area under the curves is still very high at 0.995 and 0.994, respectively. This result implies that even in the worst case, the proposed system correctly identifies 994 out of 1000 bacteria. Finally, Figure 6d shows a breakdown of performance for each class in the form of a confusion matrix. Specifically, the identification of *E. coli*, *S. aureus*, and *E. faecalis* is very clear at 98% or higher, and the distinguishing of *B. subtilis* and *S. mutans* is also quite satisfactory (≥91%). These results show that the SVM algorithm combined with the green fabricated antibacterial SERS platform can be an effective platform for detecting and identifying various bacterial strains.

### 3.6 Effect of Bacteria Disintegration on the SERS Analysis

**Figure 7:**
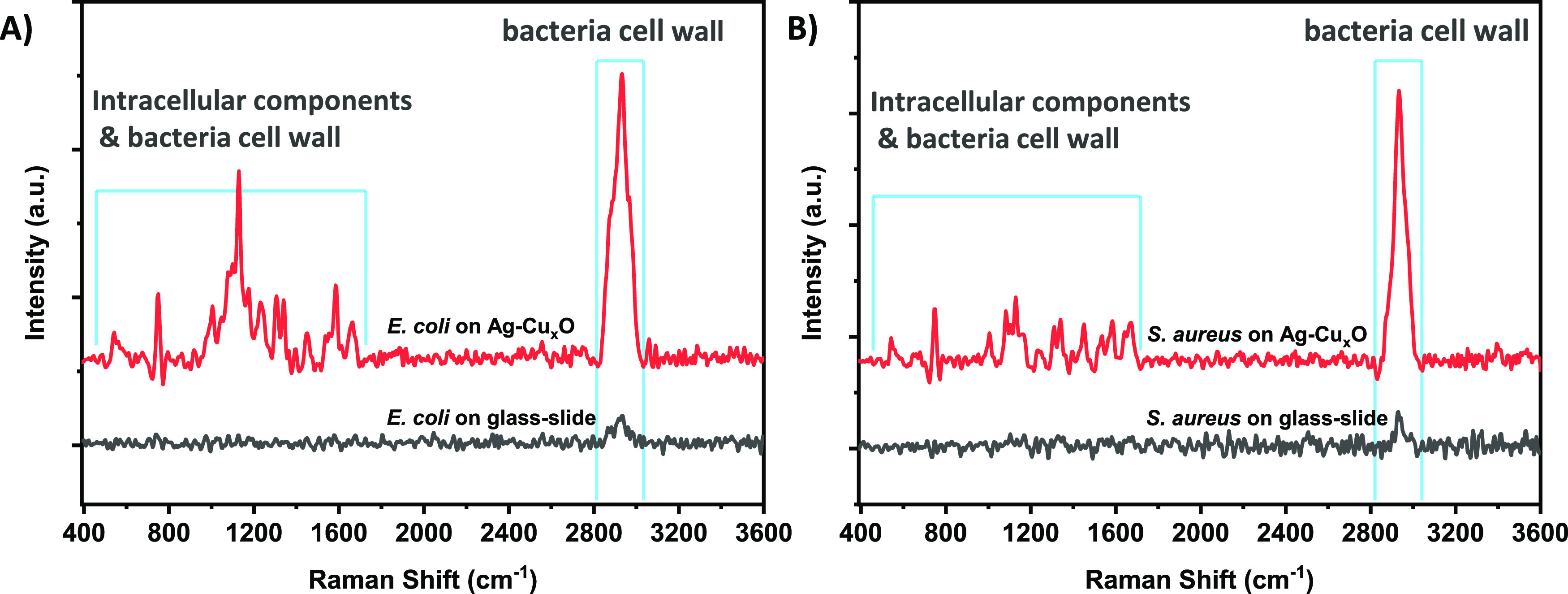
SERS spectra of bacterial strains collected on the surface of the Ag–Cu_*x*_O nanostructures, emphasizing the intracellular components. Shown are the Raman spectra of (A) Gram-negative *E. coli* and (B) Gram-positive *S. aureus* bacteria recorded on a glass slide and on the surface of the Ag–Cu_*x*_O nanostructure.

The proposed antibacterial, machine-learning-assisted SERS platform can identify multiple bacterial strains in a label-free manner with >96% accuracy. Here, we postulate that the key factor in this performance is the release of intracellular components resulting from the disintegration of bacteria. Specifically, *S. aureus* has no outer lipid membrane and contains a thick peptidoglycan layer, while *E. coli* contains a thin peptidoglycan layer and has a lipid membrane in the outermost layer. In addition to these compounds, other chemical compounds in the outer cell envelope such as polysaccharides, fatty acids, lipoproteins, and their organization on the cell surface may cause minimal changes in the spectra of bacteria.^62^ However, the intracellular and extracellular spectral characteristics of a bacterium have a deeper divergent Raman vibrational pattern due to the metabolomes that are directly related to the intracellular components.^63^ For example, Lemma et al. examined the SERS spectra of lysed and untreated *E. coli* in depth and showed that the U-T-C ring modes and the symmetrical breathing vibrations of tryptophan in the DNA/RNA bases make a difference in relation to bacterial integrity.^62^ Therefore, intracellular components may also facilitate bacterial identification by increasing the number of characteristic Raman peaks from biomolecules such as DNA, RNA, and protein in addition to cell surface components. In this direction, Allen et al. reported that the released intracellular components provided highly reproducible SERS spectra and that when a small number of isolates was evaluated, it was possible to distinguish between multiple bacterial species.^30^ However, Cui et al. emphasized that the concentration of toxic-SERS-active nanoparticles that cause leakage of intracellular components and the incubation time with bacteria cause variance in bacterial SERS spectra, thus making their use in bacterial identification challenging.^64^ Contrary to this concern, in this study, the high antibacterial activity of the surface caused bacteria to die in a very short time (Figures S6B and S13), resulting in very similar characteristics of SERS signals collected on the surface at different times (Figure S14A). Closer examination of the image taken within 1 h of bacterium cultivation showed deep cracks that indicate cell fragmentation (Figure S19). This observation supports the SERS results, where abundant characteristic peaks of intracellular components such as DNA, RNA, and extracellular leaking proteins, in addition to the peaks indicating cell membrane components were detected (Figure 7 and Table S6). Therefore, the increase in characteristic peaks that emerged with the disintegration of the bacteria enabled the high SERS sensitivity for identifying between different bacterial species. Furthermore, having reproducible spectra in the same bacterial strain and increased variance among different strains provided an advantage for machine learning and enabled bacterial identification with a high success rate. To our knowledge, although there are studies that follow the antibacterial mechanism with SERS^65−67^ or evaluate these properties separately,^68,69^ there is no study to identify bacteria by utilizing antibacterial activation. In this respect, the presented results may guide the exploitation of antibacterial activity in SERS-based bacterial identification.

## 4 Conclusions

In conclusion, this study has presented a multi-functional platform based on Ag–Cu_*x*_O nanostructures for the disintegration and identification of bacteria. The platform exhibited high lethality against Gram-positive and Gram-negative bacteria thanks to the high ion release of copper oxide nanoparticles and ROS activation of metallic nanoparticles. Additionally, the silver nanoparticles on the surface imparted SERS capability to detect five different bacterial strains with high accuracy at low cell numbers. This capability was made possible using machine learning algorithms to assist the classification of bacteria species from their SERS spectra and were able to directly identify *B. subtilis*, *E. coli*, *E. faecalis*, *S. aureus*, and *S. mutans* with >96% accuracy, without any additional processing. A key factor in the effective detection and identification of bacteria is the disintegration and release of intracellular components on the same platform. The Ag–Cu_*x*_O nanostructure appears to offer a promising solution for fighting bacterial contamination with their unique combination of antibacterial and sensing capabilities. The design approach presented in this study can establish a key point for further development through the synergetic combination of different nanoscale materials, fabrication methods, and sensing approaches.

## Supporting Information Available

Size distribution of the nanoparticles, high-resolution XPS scan around Cu 2p region and the Ag 3d region, elemental analysis of untreated print paper, photographs of agar plate showing the diffusion disk, the bactericidal activity of prepared surfaces against *E. coli* depending on time and concentration, fluorescence microscopy images showing intracellular ROS generation, SERS activity of different surfaces, additional SERS and FTIR spectra related to bacteria, reproducibility data of bacterial SERS spectra, SERS spectra of *E. coli* at various cell counts, additional data on PCA and machine-learning analysis, detailed assignment of XRD peaks, the detailed results of the antibacterial activity of the surfaces, ion release results of the surface, and detailed peaks in the SERS spectra of bacteria (PDF)
