# Supplementary material for: Disintegration and Machine-Learning-Assisted Identification of Bacteria on Antimicrobial and Plasmonic Ag–CuxO Nanostructures
Source: ACS Appl Mater Interfaces. 2023 Feb 22;15(9):11563–74. doi: 10.1021/acsami.2c22003 (PMC9999350; doi:10.1021/acsami.2c22003)
Supplement: Supplementary file 1 — am2c22003_si_001.pdf [file am2c22003_si_001.pdf]

Supporting Information

for

Disintegration and Machine Learning Assisted Identification of Bacteria on Antimicrobial and Plasmonic Ag-Cu_xO Nanostructures

Furkan Sahin [1], *Ali Camdal* [2], *Gamze Demirel Sahin* [3], *Ahmet Ceylan* [4], *Mahmut Ruzi* [1], *Mustafa Serdar Onses* [1,5,6*]

[1] ERNAM - Erciyes University Nanotechnology Application and Research Center, Kayseri, 38039, Turkey

[2] Department of Electronic Engineering, Trinity College Dublin, Dublin 2 College Green, Dublin 2, Ireland

[3] Department of Biomedical Engineering, Yildiz Technical University, Istanbul, 34220, Turkey

[4] Faculty of Pharmacy, Erciyes University, Kayseri, 38039, Turkey

[5] Department of Materials Science and Engineering, Erciyes University, Kayseri, 38039, Turkey

[6] UNAM−Institute of Materials Science and Nanotechnology, Bilkent University, Ankara, 06800, Turkey

*Address correspondence to: onses@erciyes.edu.tr (MSO)

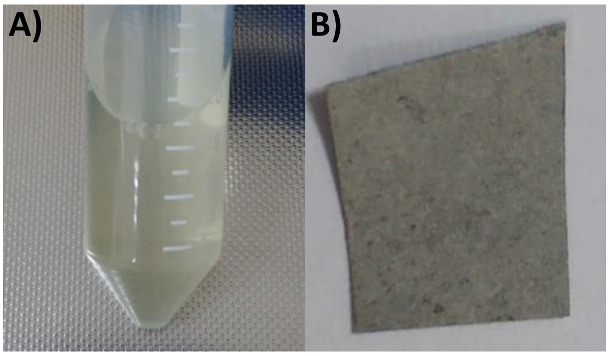

Figure S1. A photograph of A) the aqueous extract of the *C. libani*, and B) Ag-Cu_xO nanostructures grown on the paper surface.

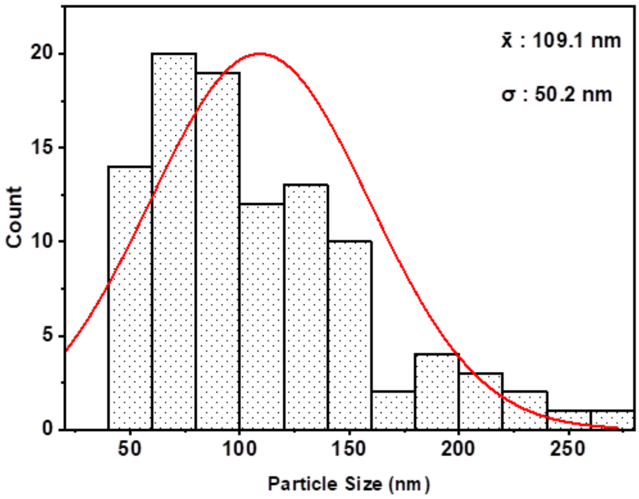

Figure S2. Size distribution of particles (n=100) on the surface according to SEM images.

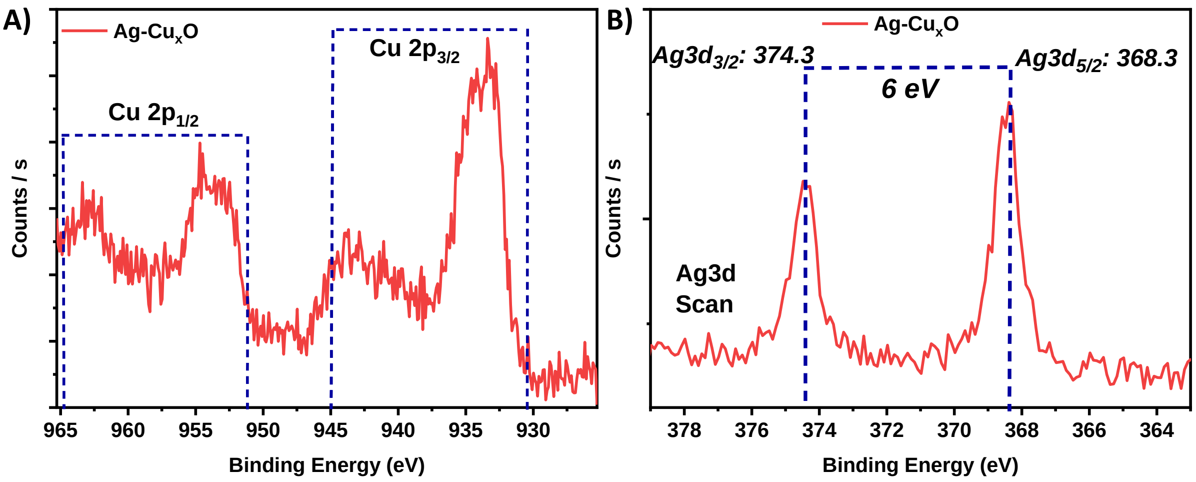

Figure S3. High-resolution XPS scan around; A) Cu 2p region, and B) Ag 3d region.

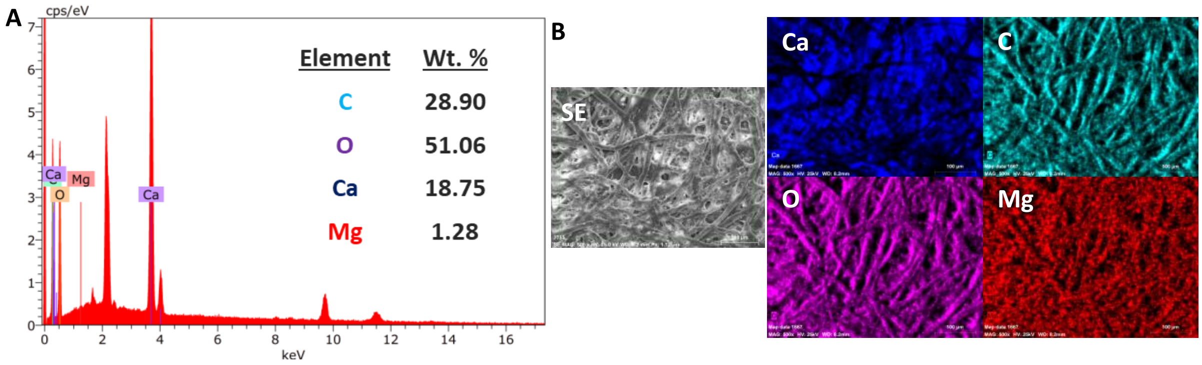

Figure S4. EDX Analysis of the untreated print paper. A) Elemental analysis and B) mapping images for different elements.

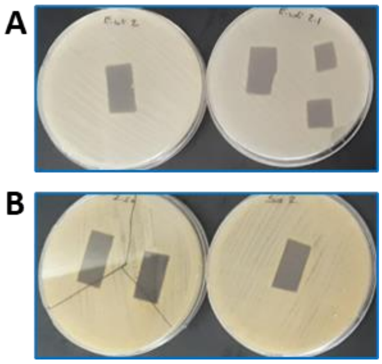

Figure S5. Antibacterial activity of the copper-free surface, which is composed of only silver. Photographs of agar plate showing the diffusion disk results for A) *E. coli*, and B) *S. aureus.* Shown are large (1×3 cm^2) and small (1×1.5 cm^2) samples.

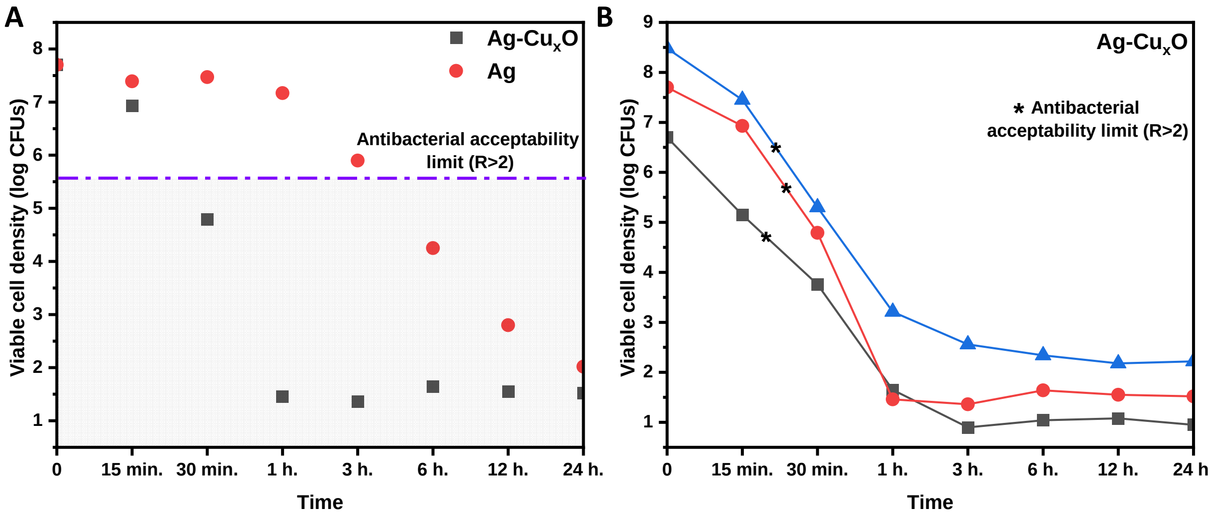

Figure S6. Evaluation of the bactericidal activity of the prepared surfaces against *E. coli*. A) Time-dependent bactericidal activity of Ag and Ag-Cu$_x$O. B) Bactericidal activity of Ag-Cu$_x$O against bacterial suspensions of different concentrations. The starting cell counts for the blue, red, and black lines are 3×10^8, 5×10^7, and 5×10^6 CFU/mL, respectively. The violet line in part A and * mark in part B show the antibacterial activity limit for each concentration as determined by 10^2 fold reduction in the number of cells.

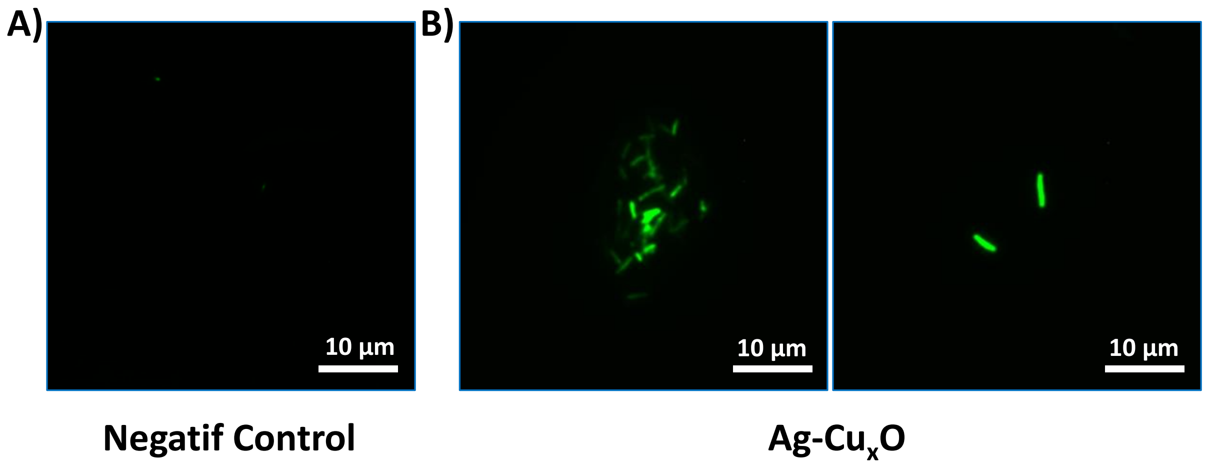

Figure S7. Fluorescence images of DCFH-DA (green emission, λ_{em} = 523 nm) stained *E. coli*. cultivated on A) the control sample and B) Ag-Cu_xO sample. Here, the images are taken from two different positions. The ROS marker DCFH-DA fluoresces upon oxidation, indicating the presence of intracellular ROS generation.

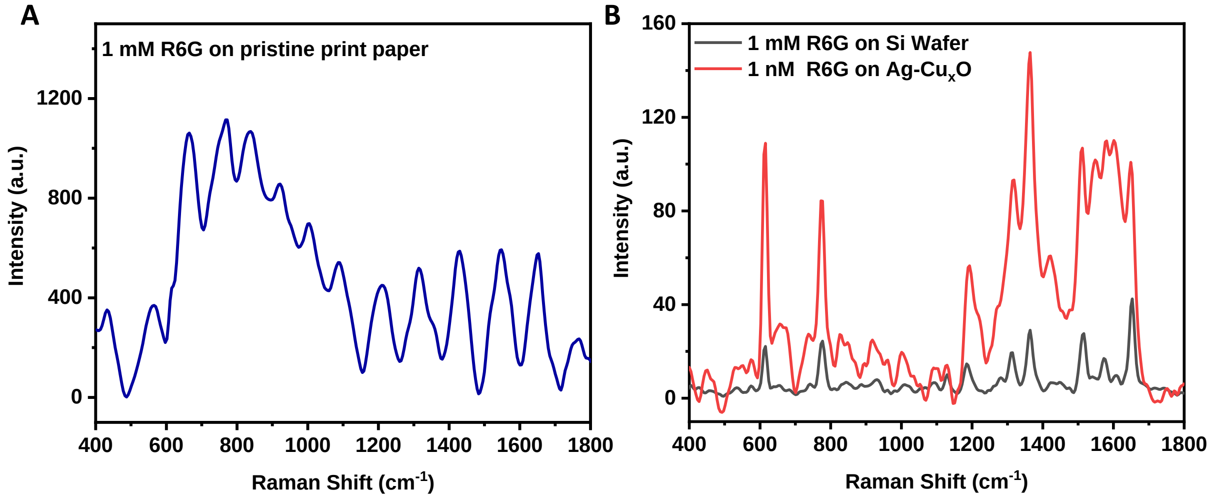

Figure S8. SERS spectrum of rhodamine 6G collected on A) untreated paper, B) silicon wafer (black), and the prepared Ag-Cu_xO nanocomposite surface.

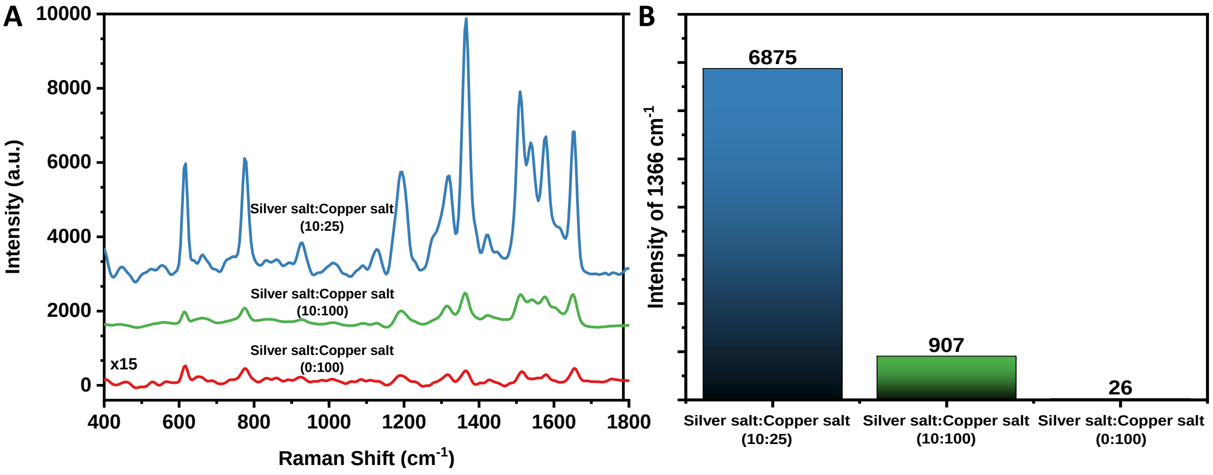

Figure S9. Effect of the ratio (W:W) of silver salt: copper salt on the SERS activity. A) SERS spectra of R6G (100 μM) collected from surfaces of 10:25 (blue, Ag-Cu_xO), the 10:100 (green), and 0:100 (red) of silver salt : copper salt. B) The intensity of R6G at a position of 1366 cm^{-1} for different ratios of silver salt : copper salt.

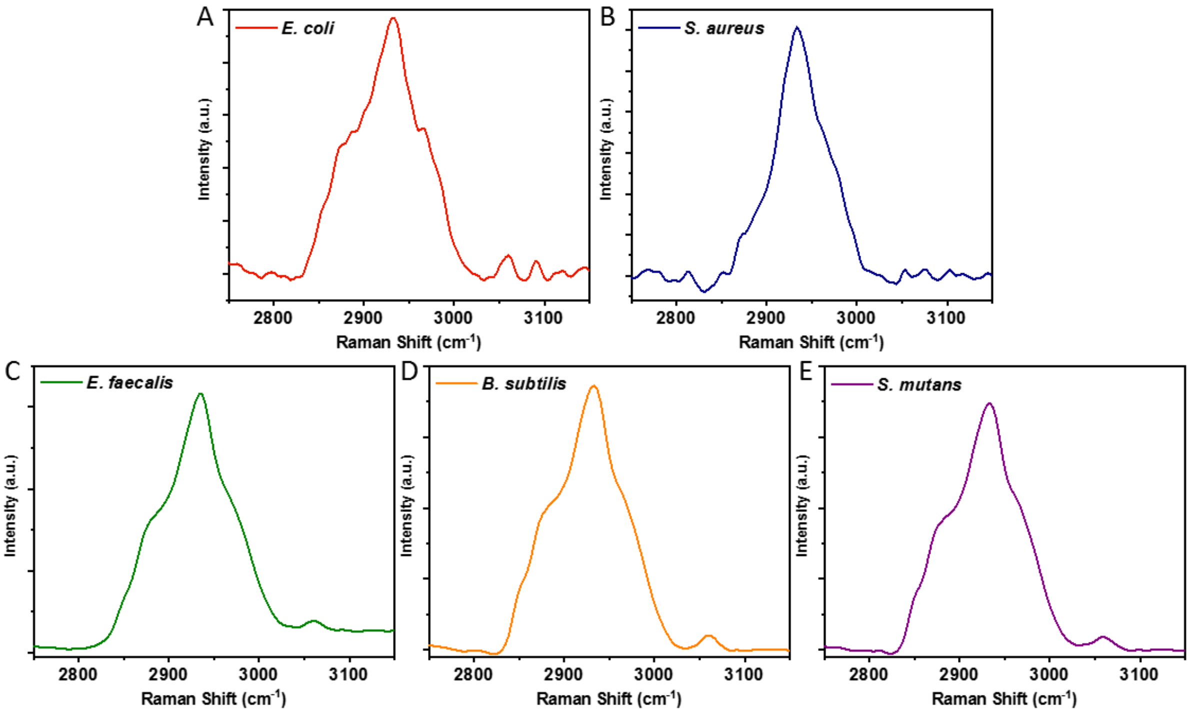

Figure S10. The SERS spectra of bacteria species; A) *E. coli*, B) *S. aureus*, C) *E. faecalis*, D) *B. subtilis* and E) *S. mutans* around the CH stretch region.

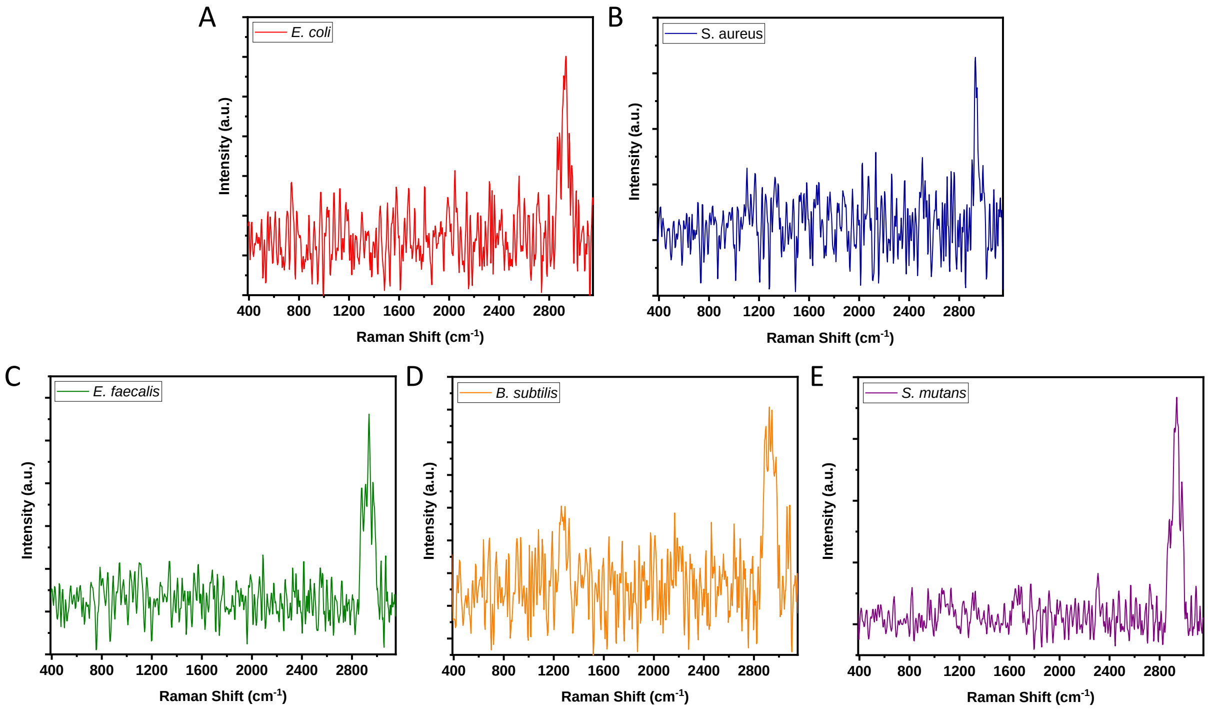

Figure S11. Raman spectra of various bacteria species collected on glass slides. A) *E. coli*, B) *S. aureus*, C) *E. faecalis*, D) *B. subtilis* and E) *S. mutans*.

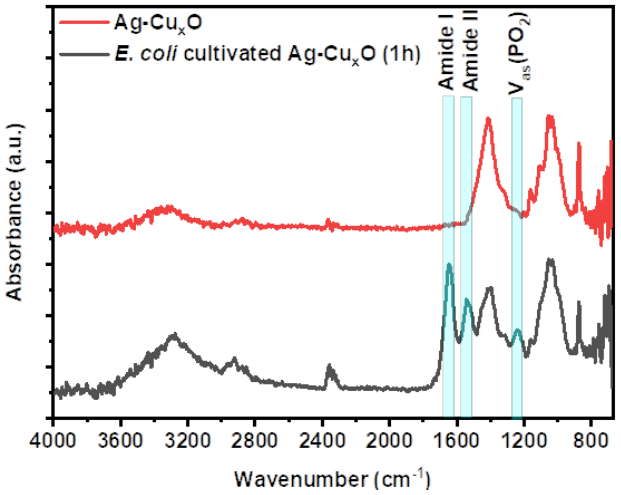

Figure S12. FTIR spectrum of the Ag-Cu$_x$O nanocomposite and *E. coli* cultivated Ag-Cu$_x$O.

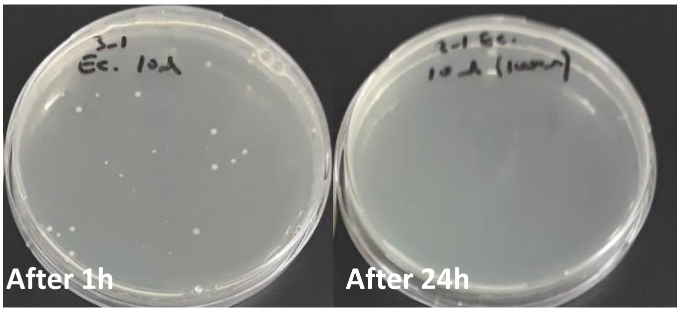

Figure S13. Antibacterial activity of the surface against *E. coli* after 1 h and 24 h.

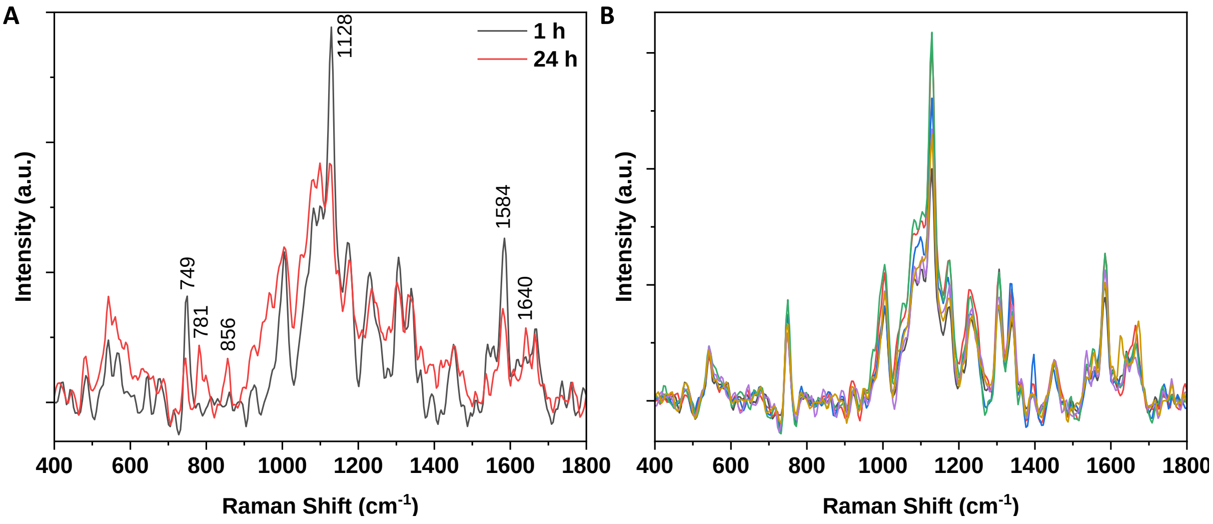

Figure S14. Reproducibility of bacteria SERS spectra collected on Ag-Cu$_x$O nanostructures. A) SERS signals collected from the surface after *E. coli* inoculation for 1 h and 24 h. B) SERS signals collected from 6 different regions of the surface after 1 h.

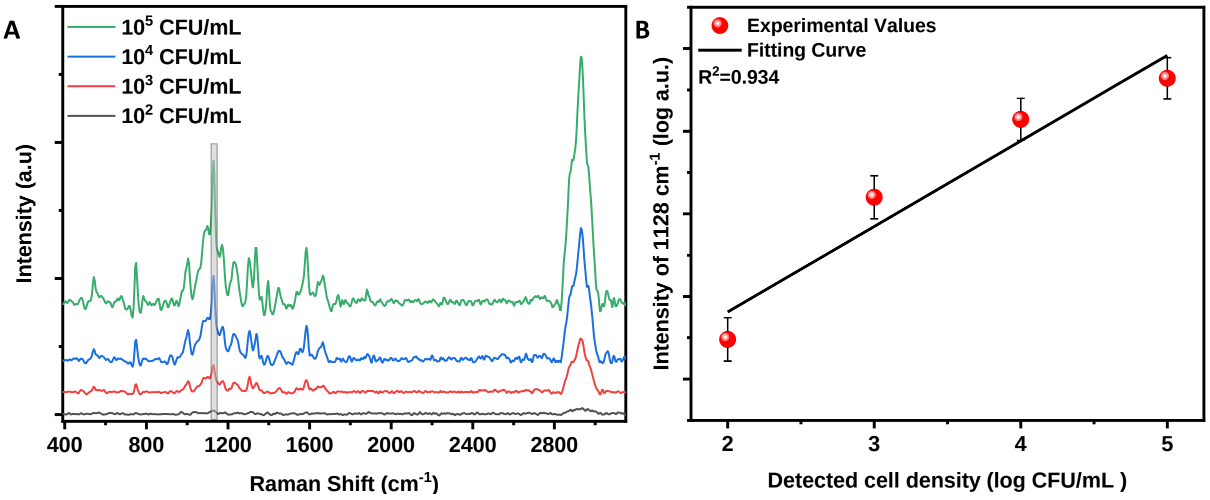

Figure S15. A) SERS spectra of *E. coli* at various cell counts. B) The SERS intensity of the peak at 1128 cm^{-1} as a function of the concentration of *E. coli*.

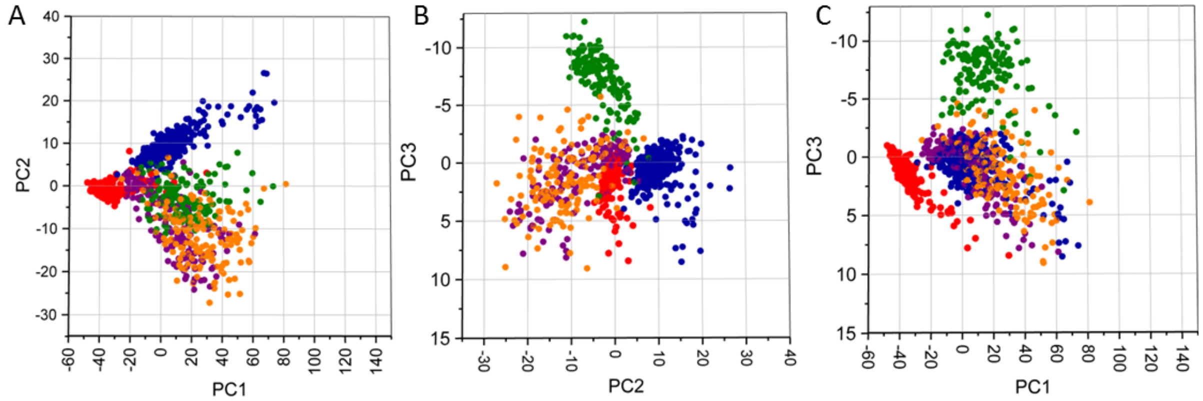

Figure S16. PCA results in 2D space. A) PC1-PC2 plane, B) PC2-PC3 plane, C) PC1-PC3 plane.

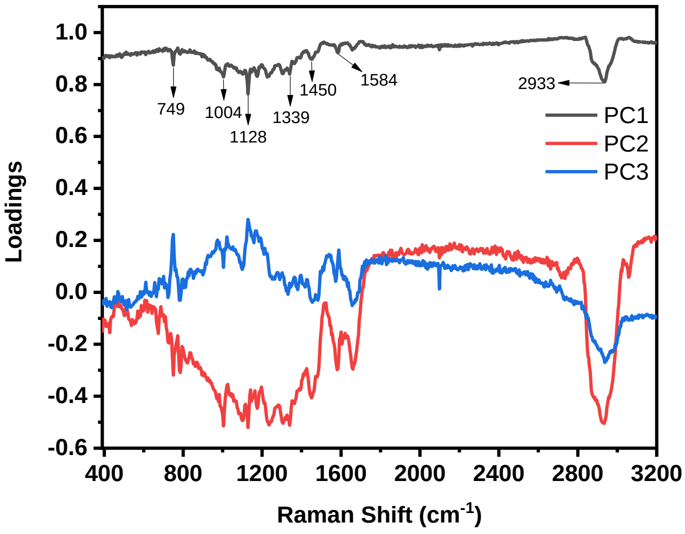

Figure S17. Loading plots of the three components represent 84% of the total variance.

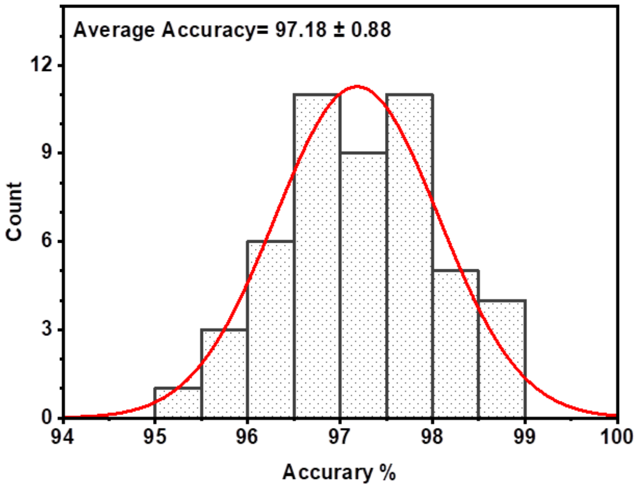

Figure S18. The distribution of the accuracy rates obtained as a result of training the SVM (linear) model with 50 different data sets (randomization coefficient=40).

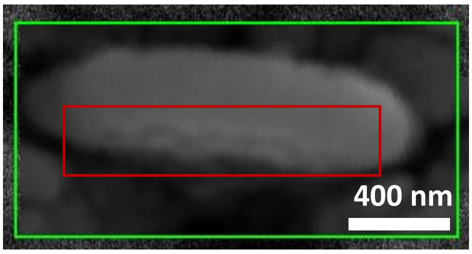

Figure S19. SEM image of *E. coli* on the nanocomposite at 100,000× magnifications. The image shows a deep crack in *E. coli*.

Table S1. The production cost of the Ag-Cu_xO nanocomposite.

Product	Unit Price	Price of quantity used
Deionized Water	$0.54 / L	$0.008 / 15mL
Filter Paper	$4 / pack of 100	$0.04 / pack of 1
Falcon Conical Tube (50 ml)	$0.08 / piece	$0.08 / piece
Copy Paper	$0.009 / sheet (210 x 297 mm, 80 g/m2)	$0.00004 / a piece of (10 x 30 mm)
Silver Nitrate	$490 / 250 g	$0.02 / 10 mg
Copper (II) acetate	$150.00 / 250 g	$0.015 / 25 mg
**Total Price**		**$0.16**

Table S2. Detailed assignment of XRD peaks(2θ) and planes of components.

Compound	XRD Peaks(2θ)	Planes
Ag	38, 44, 65, 77 and 82°	(111), (200), (220), (311) and (222)
CuO	32.6, 35.7, 48.8, 53.4, 58.2, 61.6, 65.8, 66.2, and 68.1°	(110), (002, $\bar{1}11$), ($\bar{2}02$), (020), (220), ($\bar{1}13$), (022), ($\bar{3}11$), and (220)
Cu_2O	61.51, 73.75, and 77.53°	(220), (311), and (222)
$CaCO_3$	23.2, 29.5, 31.5, 36, 39.5, 43.2, 47.6, 48.6, 56.6, 57.5, and 60.9°	(102), (104), (006), (110), (113), (202), (018), (116), (211), (122), and (208)
Cellulose	16.1 and 22.6°	(110) and (200)

Table S3. The results of the inhibition of the Ag-Cu_xO nanocomposite surface against *E. coli* and *S. aureus*.

Sample name	Diffusion Distance After 24 Hours	
	E. coli	*S. aureus*
**Ag**	0.79 ± 0.32 mm	0
**Ag-Cu_xO**	2.67 ± 0.47 mm	5.67 ± 0.94 mm

Table S4. The number of living colonies on various surfaces after 24 h.

Sample name	Number of initial colonies (CFU/ml)		Colony Units After 24 hours (CFU/ml, n=4)		Antibacterial Activity (24h) R=log(Control)-log(Sample)	
	E. coli	*S. aureus*	*E. coli*	*S. aureus*	*E. coli*	*S. aureus*
**Paper**	2.5×10^5	5.6×10^5	5×10^7	9×10^7	-	-
**Ag**	2.5×10^5	5.6×10^5	105 ± 36	100 ± 28	5.68 ± 0.14	5.95 ± 0.12
**10:25**[a]	2.5×10^5	5.6×10^5	33 ± 8.5	52 ± 30	6.18 ± 0.11	6.24 ± 0.27
**10:100**[a]	2.5×10^5	5.6×10^5	6 ± 2	12 ± 6	6.92 ± 0.13	6.95 ± 0.21

a: indicates the mass ratio of silver salt to copper salt. For example, in the Ag-Cu_xO sample, the ratio is 10 mg:25 mg.

Table S5. The number of leached Ag and Cu ions at different times from the Ag-Cu_xO sample immersed in deionized water. The measurement was performed using ICP-MS

The type of ions	The number of ions leached (ppm)	
	1 h	***24 h***
**Ag**	0.386	0.456
**Cu**	1.625	1.934

Table S6. Detailed assignment of the peaks in the SERS spectra of bacteria. Note: υ means stretching, δ is in-plane bending and γ means out-of-plane bending. Note: υ means stretching, δ is in-plane bending and γ means out-of-plane bending. Abbreviations: A, adenine; G, guanine; T, thymine; C, cytosine; Tyr, tyrosine; Phe, phenylalanine; Trp, tryptophan; Lys, lysine; Val, valine

E. coli	***S. aureus***	***E. feacalis***	***B. subtilis***	***S. mutans***	**Assignment**	**Origin**	***Reference***
		422	422	427	υ(C–C)	Skeletal modes of carbohydrates	1,2
446	446		441	446	δ(CCC) ring deformation	Carbohydrates	2
487	478		478	478	ν(CC) backbone	Carbohydrates	2
	492		496	496			
542	542	542	542	542	υ(S–S), symmetric skeletal vib.	Proteins, Lys, Carbohydrates	2,3
610					Breathing vibration	Benzene ring	4
		624	624	619	Skeletal vibration, δ(C–C)	Aromatic ring, Phe, A	1,4,5
642	646	642	642	642	δ(COO-)	Tyr, C	1,4–6
673	678	669	673	669	δ(COO-)	T, A	6,7
		723	718	714	C–H rocking of CH2	A, RNA, glycosidic ring mode, DNA	1,4,5,7
749	749	758	749	749	Ring breathing	Trp, T	1,3,6
794	785	785	785	785	υ(O–P–O), ring breathing	DNA, C, T	4,6–8
	816	812	816	816	υ(CN), υ(O–P–O)	Lipids, Tyr, Porin, Val,	1,5–7

						RNA	
860	860	856	856	856	υ(C–C), ring breathing	Tyr, Deoxyribose-phosphate groups	1,3,6–8
891	891	900	886	891	υ(C–C)	Lecithin	2
917	930	935	921	921	υ(COO–), υ(C–C)	Skeletal proteins, ring deformation of A	1,4,6–8
	952		978	974	υ(C–N), C=C deformation	Lipids	1,2,4,5
1004	1004	1004	1004	1004	C–C aromatic ring breathing	Phe	3,4,7
1047	1047	1039	1034	1039	C–C ring breathing, υ(C–O)	Phe, Thiamine	1,4,6,8
1077	1081	1073			υ(PO^{-2})	DNA/RNA	8
1099	1103	1099	1103	1099	υ(C–O), υ(C–C), υ(–C–OH), O–P–O-	Carbonhydrates, DNA	4,6,7
1128	1128	1124	1128	1128	υ(C–N), υ(C–C), υ(C–O–C)	Amide III, A, Phe	1,4,5,7
1175	1162	1175	1175	1175	υ(C–C, C–H)	C, T	4,6,8
1230	1230		1234	1234	υ(C–C), δ(C–C)	Amide III	1,4,6,8
	1247	1247			δ(CH2), υ(C–C), δ(C–C)	Amide III, Amide I, C, A	1,3–7
1305	1310	1310	1305	1305	−C–H def	A, protein	4,6
1339	1339	1339	1339	1339	−CH deformation, υ(NH2), δ(C–H)	A, G, DNA, Trp	1,4,6,7,9
1364		1376			δ(C-H), υ(C–N), υ(COO-) δ(C–C)	Phenyl ring, G, T, A, protein	1,4,6,8

1397	1397		1393	1393	δ(CH)	T	8
1450	1450	1450	1450	1450	−CH2 deformation, δ(CH2)	Proteins, saturated lipids, Trp	1,3–5,7,8
1536	1536				υ(CN), δ(NH)	amide II	1
1552					CH_2 deformation	Guanine	4,8
1584	1584	1576	1580	1580	Ring strech	A, G, Tyr	1,4,6–8
1612			1608-1620	1608	C=C, δ(NH_2)	Phe, Tyr, Amide I, Unsaturated lipids, A, G, C	1,4,6,7
	1640				υ(CN), δ(NH)	Amide II	5
1664	1668	1664	1660	1660	υ(C=O), υ(C=N), δ(NH_2)	Amide I, T, C	5–7
1720	1736				υ(C=O)	Amide I α-helix	4
1747	1747		1740	1747	υ(C=O)	Saturate esters	2
1763	1759		1759	1763	C_2=O	T	8
	2810				υ(C–H)	proteins and lipids	10
2886	2872	2882	2879	2879	υ(C–H)	proteins and lipids	10
2933	2933	2933	2933	2933	υ(C–H)	proteins and lipids	10
3060	3053	3060	3060	3060	υ(C–H)	hetero aromatics	11
3090	3076				υ(C–H)	hetero aromatics	11

REFERENCES

(1) Liu, Y.; Zhou, H.; Hu, Z.; Yu, G.; Yang, D.; Zhao, J. Label and Label-Free Based Surface-Enhanced Raman Scattering for Pathogen Bacteria Detection: A Review. *Biosens Bioelectron* **2017**, *94*, 131–140. https://doi.org/10.1016/J.BIOS.2017.02.032.

(2) de Gussem, K.; Vandenabeele, P.; Verbeken, A.; Moens, L. Raman Spectroscopic Study of Lactarius Spores (Russulales, Fungi). *Spectrochim Acta A Mol Biomol Spectrosc* **2005**, *61* (13–14), 2896–2908. https://doi.org/10.1016/J.SAA.2004.10.038.

(3) Virkler, K.; Lednev, I. K. Raman Spectroscopy Offers Great Potential for the Nondestructive Confirmatory Identification of Body Fluids. *Forensic Sci Int* **2008**, *181* (1–3), e1–e5. https://doi.org/10.1016/J.FORSCIINT.2008.08.004.

(4) Bashir, S.; Ali, S.; Nawaz, H.; Majeed, M. I.; Mohsin, M.; Nawaz, A.; Rashid, N.; Tahir, F.; Haq, A. ul; Saleem, M.; Nawaz, M. Z.; Shahzad, K. Characterization of Tigecycline-Sensitive and Tigecycline-Resistant Escherichia Coli by Surface-Enhanced Raman Spectroscopy (SERS) and Chemometrics. *https://doi.org/10.1080/00032719.2022.2030349* **2022**, *55* (11), 1833–1845. https://doi.org/10.1080/00032719.2022.2030349.

(5) Zhou, H.; Yang, D.; Ivleva, N. P.; Mircescu, N. E.; Niessner, R.; Haisch, C. SERS Detection of Bacteria in Water by in Situ Coating with Ag Nanoparticles. *Anal Chem* **2014**, *86* (3), 1525–1533. https://doi.org/10.1021/AC402935P/SUPPL_FILE/AC402935P_SI_001.PDF.

(6) Ke, W.; Yu, D.; Wu, J. Raman Spectroscopic Study of the Influence on Herring Sperm DNA of Heat Treatment and Ultraviolet Radiation. *Spectrochim Acta A Mol Biomol Spectrosc* **1999**, *55* (5), 1081–1090. https://doi.org/10.1016/S1386-1425(98)00225-X.

(7) Xie, C.; Mace, J.; Dinno, M. A.; Li, Y. Q.; Tang, W.; Newton, R. J.; Gemperline, P. J. Identification of Single Bacterial Cells in Aqueous Solution Using Confocal Laser Tweezers Raman Spectroscopy. *Anal Chem* **2005**, *77* (14), 4390–4397. https://doi.org/10.1021/AC0504971.

(8) Dastgir, G.; Majeed, M. I.; Nawaz, H.; Rashid, N.; Raza, A.; Ali, M. Z.; Shakeel, M.; Javed, M.; Ehsan, U.; Ishtiaq, S.; Fatima, R.; Abdulraheem, A. Surface-Enhanced Raman Spectroscopy of Polymerase Chain Reaction (PCR) Products of Rifampin

Resistant and Susceptible Tuberculosis Patients. *Photodiagnosis Photodyn Ther* **2022**, *38*, 102758. https://doi.org/10.1016/J.PDPDT.2022.102758.

(9) Zhou, H.; Yang, D.; Ivleva, N. P.; Mircescu, N. E.; Niessner, R.; Haisch, C. SERS Detection of Bacteria in Water by in Situ Coating with Ag Nanoparticles. *Anal Chem* **2014**, *86* (3), 1525–1533. https://doi.org/10.1021/AC402935P/SUPPL_FILE/AC402935P_SI_001.PDF.

(10) Jamieson, L. E.; Wetherill, C.; Faulds, K.; Graham, D. Ratiometric Raman Imaging Reveals the New Anti-Cancer Potential of Lipid Targeting Drugs. *Chem Sci* **2018**, *9* (34), 6935–6943. https://doi.org/10.1039/C8SC02312C.

(11) Howell, N. K.; Arteaga, G.; Nakai, S.; Li-Chan, E. C. Y. Raman Spectral Analysis in the C−H Stretching Region of Proteins and Amino Acids for Investigation of Hydrophobic Interactions. *J Agric Food Chem* **1999**, *47* (3), 924–933. https://doi.org/10.1021/JF981074L.
